# Comparative transcriptomic analysis indicates genes associated with local and systemic resistance to *Colletotrichum graminicola* in maize

**DOI:** 10.1038/s41598-017-02298-8

**Published:** 2017-05-30

**Authors:** Vívian de Jesus Miranda, William Farias Porto, Gabriel da Rocha Fernandes, Robert Pogue, Diego Oliveira Nolasco, Ana Claudia Guerra Araujo, Luciano Viana Cota, Camila Guimarães de Freitas, Simoni Campos Dias, Octavio Luiz Franco

**Affiliations:** 1 Centro de Análises Proteômicas e Bioquímicas, Pós-Graduação em Ciências Genômicas e Biotecnologia Universidade Católica de Brasília, Brasília-DF, Brazil; 2 Porto Reports, Brasília, DF Brazil; 3 Fundação Oswaldo Cruz, Fiocruz, Belo Horizonte Brazil; 4 Research Laboratory of Electronics - Massachusetts Institute of Technology (MIT), Cambridge, MA USA; 5 Embrapa Recursos Genéticos e Biotecnologia, Brasília, DF Brazil; 6 Embrapa Milho e Sorgo, Sete Lagoas, Brazil; 7 Instituto Federal de Brasília, DF, Brazil; 8 S-Inova Biotech, Pos-Graduação em Biotecnologia, Universidade Católica Dom Bosco, Campo Grande, Brazil

## Abstract

The hemibiotrophic fungus *Colletotrichum graminicola* may cause severe damage to maize, affecting normal development of the plant and decreasing grain yield. In this context, understanding plant defense pathways at the inoculation site and systemically in uninoculated tissues can help in the development of genetic engineering of resistance against this pathogen. Previous work has discussed the molecular basis of maize - *C. graminicola* interaction. However, many genes involved in defense have not yet been exploited for lack of annotation in public databases. Here, changes in global gene expression were studied in root, male and female inflorescences of maize under local and systemic fungal infection treatments, respectively. RNA-Seq with qPCR was used to indicate genes involved in plant defense. We found that systemic acquired resistance induction in female inflorescences mainly involves accumulation of salicylic acid (SA)-inducible defense genes (*ZmNAC*, *ZmHSF*, *ZmWRKY*, *ZmbZIP* and *PR1*) and potential genes involved in chromatin modification. Furthermore, transcripts involved in jasmonic acid (JA) and ethylene (ET) signaling pathways were also accumulated and may participate in plant immunity. Moreover, several genes were functionally re-annotated based on domain signature, indicating novel candidates to be tested in strategies involving gene knockout and overexpression in plants.

## Introduction

The plant pathogenic fungus *Colletotrichum graminicola* (Ces.) G.W. Wils. is the causal agent of maize anthracnose stalk rot and leaf blight. This disease is an economically important problem that causes a worldwide impact on maize production, with annual losses of up to 1 billion dollars in the USA^1, 2^. The fungus can infect all plant parts and can be found throughout the growing season^3^. In roots, infection patterns differ from those in the leaf, because the epidermal and cortical cells are infected in a mosaic pattern, different from the cell-cell spread of primary hyphae observed in leaves. Leaf symptoms appear around three days after inoculation (d.a.i), but in the roots, no symptoms may occur up to 42 d.a.i^2, 4^. An important discovery in this pathosystem was that *C. graminicola* (*Cg*) can colonize the root, enter the vascular system and spread systemically to the aerial parts of the plant without causing widespread disease symptoms^4^.

The molecular basis of the interaction between maize and *C. graminicola* has been investigated. In this context, the genome of *C. graminicola* was published in 2012, along with transcriptomic analysis of the fungus grown *in vitro* and *in planta*, in order to clarify the mechanisms of fungal infection^5^. Additionally, as regards the plant itself, the local and systemic defense responses during fungal infection have been studied. Initially, the localized acquired resistance (LAR) in leaves infected by *C. graminicola* was evaluated with histochemical, biochemical and transcriptional analysis in the same place as the inoculation was performed^6^. It was found that this hemibiotrophic pathogen does not suppress plant defenses during the biotrophic phase, and there is an increase in defense gene expression (including PR1, PR5, chitinases and glucanases) with the progress of infection^6^. Subsequently, the systemic acquired resistance (SAR) was studied in leaf and root of maize infected by *C. graminicola*. It was found that leaf and root inoculated by *C. graminicola* have the ability to activate the systemic antifungal resistance in distal uninoculated tissues of the plant, and this signaling is involved with accumulation of salicylic acid (SA) and abscisic acid (ABA), increasing systemic resistance against secondary *C. graminicola* infection^2^. However, these reports^2, 6^ did not explore the global maize transcriptome and did not report the involvement of peptides in plant defense.

Plants have a complex array of defense mechanisms that act against pathogen attack, involving structural and chemical barriers and the production of inducible defense-related proteins (PR proteins)^7^. PR proteins are a component of Pathogen-Associated Molecular Pattern (PAMP)-triggered immunity (PTI) and may act as flags for systemic defense or can directly combat pathogenic invasion. Previously, 17 families of PR proteins were reported, and they involve members with different functions such as chitinases (PR3, PR4, PR8 and PR11), β−1,3-glucanase (PR2), osmotin and thaumatin-like protein (PR5), RNase (PR-10), defensin (PR12), thionin (PR13), lipid-transfer protein (PR14) and oxalate oxidase (PR15 and 16)^7^. Within this group of defense-related proteins many classes of antimicrobial peptides (AMPs) are highlighted due to their biotechnological potential. Plant AMPs are mostly cysteine-rich, are of small size (less than 100 amino acids) and present several antimicrobial activities, such as antifungal, antibacterial and antiviral^8, 9^. However, gene expression levels of AMPs in plant are basal and not always regulated by pathogen attack^10^. Some AMPs are involved in normal plant development, in host defense against abiotic stress and frequently require an over-expression in transgenic plants to be effective in pathogen control^11, 12^.

Besides the ability to activate local defense response after recognition of PAMPs, plants emit systemic mobile signals to non-colonized tissues, activating a primed state of heightened alert, enabling quick and strong defense reaction to pathogen attack compared to native, unprimed plants^13^. In dicot plants, SAR signaling involves the accumulation of SA and SA-associated gene transcripts in the systemic uninfected tissues during the establishment of SAR^14, 15^. Little is known about signaling pathways involved in SAR activation in monocots. Previous work has reported that primed state activation in plants involves chromatin modifications, and these changes can be passed to the next generations of primed plants, allowing rapid accumulation of transcripts of defense-related genes and increased resistance to novel pathogenic infections^16, 17^. These events have been more studied in plant-bacteria interactions because the genomes/transcriptomes of phytopathogenic bacteria were obtained first^18, 19^. However, in the maize-*C. graminicola* pathosystem little is known about gene signaling pathways controlling primed state activation, and nothing is known about the involvement of AMPs in this context.

Here, the involvement of AMPs in root LAR and inflorescence SAR against *C. graminicola* infection was investigated by *in silico* and *in vitro* techniques. Defense signaling of maize activated locally and systemically was analyzed by an RNA-Seq approach, aiming to understand the network of differentially expressed genes involved in the activation of antifungal response. Regulatory components involved in antifungal protection are important tools in the development of engineering of resistance in plants^20^.

## Results

### Establishment of anthracnose disease in maize

**Figure 1:**
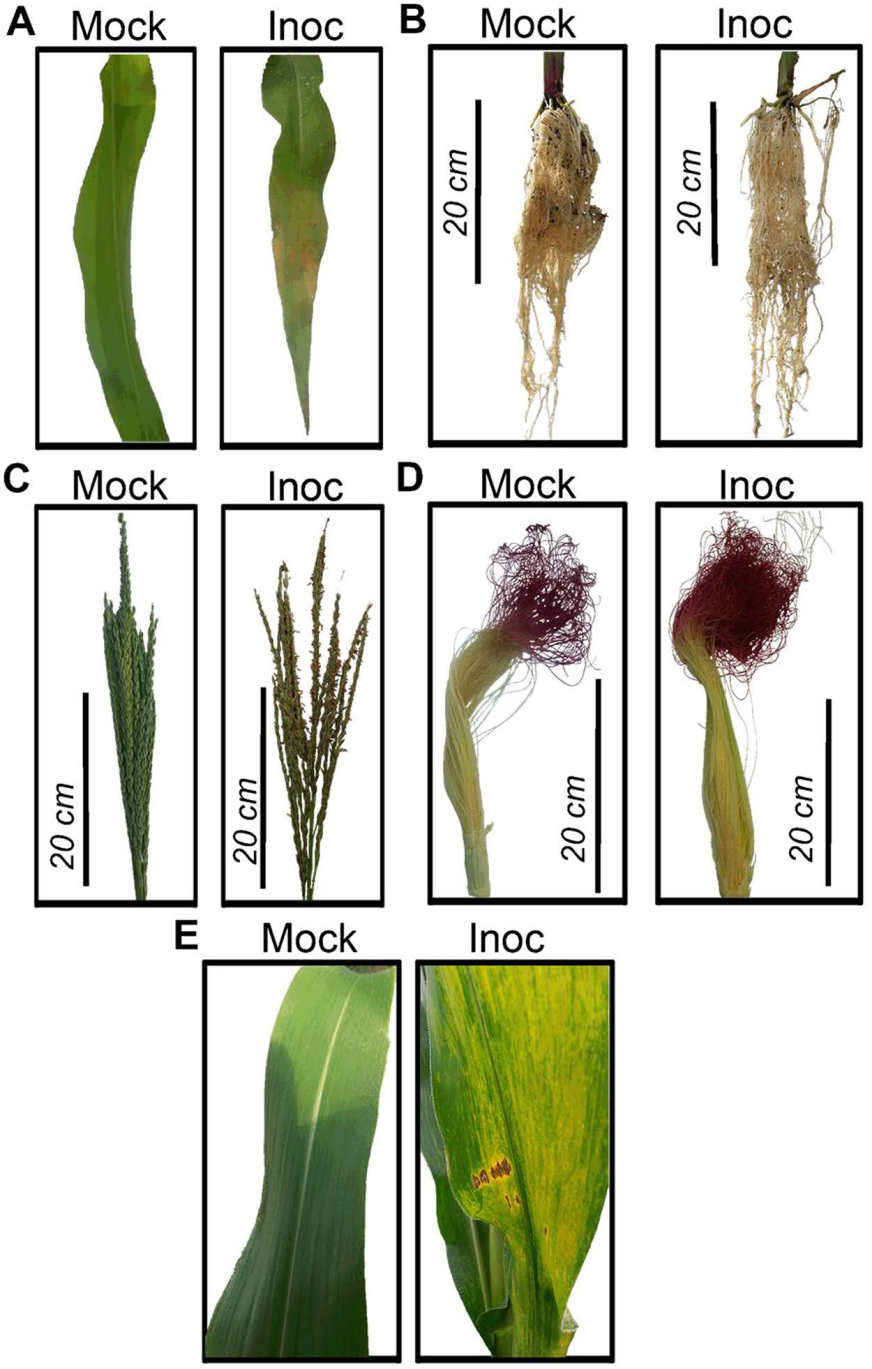
Local and systemic symptoms of anthracnose in Maize. Mock and inoculated (Inoc) maize leaf after 72 h.a.i (**A**). No symptom of disease was observed in inoculated roots 7 d.a.i (**B**). Male inflorescence showing clear symptoms of senescence in inoculated plants (**C**). A small increase in the development of inoculated female inflorescence can be observed compared to mock-plants (**D**). Maize plants in R1 stage showing anthracnose symptoms in inoculated leaves compared to mock-plants (**E**).

**Figure 2:**
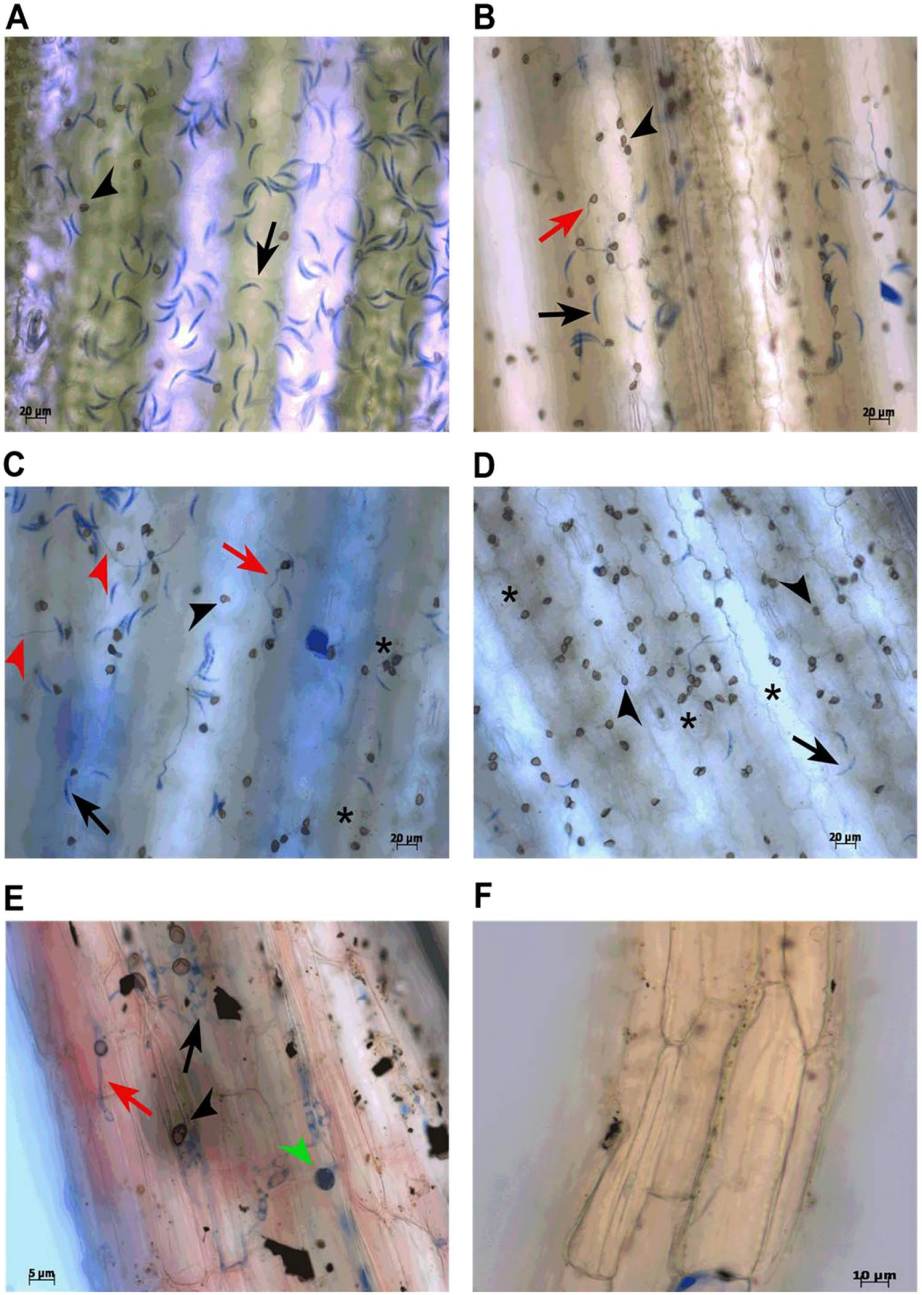
Light microscopy in bright field of leaves and root infected with *C. graminicola*. Samples were cleared and stained with lactophenol cotton blue. Leaves at 24 h.a.i (**A**), 36 h.a.i (**B**), 48 h.a.i (**C**) and 72 h.a.i *with C. graminicol*a (**D**). Individual mock treatments for each time-point are shown in Supplementary Figs S1, S2 and S3. Root at 48 h.a.i (**E**) and a mock-root section (**F**). Black arrow: spore; black arrowheads: melanized appressorium; green arrowheads: non-melanized appressorium; red arrow: primary hyphae; heads of red arrow: secondary hyphae; asterisk: hypersensitive response. All panels represent results found in three independent biological replicates.

In order to establish *C. graminicola* local infection in leaf and root of maize, light microscopy analyses were performed to ensure the disease progress in our environmental conditions. On leaves inoculated in the V4 developmental stage of maize, local symptoms began to appear 3 days after inoculation (d.a.i) (Fig. 1A). In roots, in the same period, no local symptom was observed 7 d.a.i (Fig. 1B). In systemic infections, maize plants in the R1 stage were leaf and root-inoculated with *C. graminicola*. At this stage, symptoms began to appear in leaves 12 d.a.i (Fig. 1E). When early symptoms of anthracnose began to appear in inoculated leaves, uninoculated male and female inflorescences in systemic response (SAR^+^) were collected for gene expression analysis. Phenotypically, the first indication of inflorescence defense activation in response to *C. graminicola* infection was an increased senescence rate observed in inoculated plants compared with mock plants (Fig. 1C) and increased organ development (Fig. 1D). On leaves, the adhesion of conidia occurs on the leaf epidermis surface 24 h after inoculation (h.a.i), germination and early penetration less often (Supplementary Fig. S1). Most conidia were still not germinated in these samples (Fig. 2A). At 36 h.a.i appressorium melanization and primary hyphae growth were observed between cells featuring the biotrophic stage (Fig. 2B and Supplementary Fig. S2). At 48 h.a.i early growth of secondary hyphae occurred intracellularly (transition of biotrophic-necrotrophic stages) (Fig. 2C). Cellular responses, such as production of reactive oxygen species (ROS) (Fig. 2C and D, an asterisk) and strengthening of cell wall through the presence of lignified buds, started at 48 h.a.i, but were more strongly viewed at 72 h.a.i (Supplementary Fig. S3). At 72 h.a.i few hyphae were seen superficially, due to prevailing internal colonization by secondary hyphae featuring the necrotrophic stage (Fig. 2D). In roots at 48 h.a.i, a delay in infection was observed, compared to the leaf. Melanized appressoria, appressorium in maturation and bulbous hyphae growing between cells were seen. All these events characterize the biotrophic stage of fungal growth in the root organ. (Fig. 2E and Supplementary Fig. S4). Together, these results are consistent with previous reports, indicating a correct establishment of anthracnose in local infections of maize in our environmental conditions^2, 4, 6^.

### *In silico* identification of cysteine-rich antimicrobial peptides from the NCBI database and gene expression validation

Aiming to initiate a detailed study of AMPs in maize, to know in which maize organ they are expressed and identify genes encoding AMPs regulated by *C. graminicola* infection, a screening for the main antimicrobial peptide classes was first performed in NCBI non-redundant protein database (NR). Cysteine patterns were used for AMP identification (Supplementary Table S1), with the exception of the α-harpinins class in which the research was performed by local aligning (Blastp) against the same database. AMP classes surveyed were snakins (SNK), defensins (DEF), lipid transfer proteins (LTP), thionins (THN), heveins (HEV) and α-harpinins (MBP). Initially, 241 sequences were identified. Among them, 113 sequences have specific annotation and 128 sequences were annotated as uncharacterized or LOC protein, indicating a poor annotation of peptides in the NCBI database. Among the characterized sequences, 62 were LTPs, 15 DEFs, 9 SNKs, 2 HEV/CHI (chimerolectins), and 25 other sequences were considered as artifacts. Further analyses were carried out only with sequences without annotation in the NCBI database, considering that these could be novel antimicrobial peptides to be validated. From these, 86 sequences showed secretion signals according to Phobius prediction and were maintained in analysis. Only 45 sequences were confirmed by InterProScan as containing a characteristic domain. Furthermore, 18 sequences containing tails in N or C-terminal larger than 20 amino acid residues around the active domain were excluded from screening. According to CS-AMPPred, which ranks antimicrobial activity based on five sequence descriptors, only 9 peptides do not have putative antimicrobial activity, and all of them were removed from analysis. In this step, 18 small cysteine-rich antimicrobial peptides composed of 8 LTPs, 3 DEFs, 3 SNKs and 4 HEV/CHI were selected. However, HEV/CHIs were removed from screening because the hevein domain is fusioned with a large protein domain. Interestingly for the α-harpinin class, we found six MBP domains in addition to MBP1 (maize basic peptide 1) previously described^21^. These six repeats are located after signal peptide and in tandem with the first MBP-1, in the same precursor (Supplementary Fig. S5D). Probably one post-translational proteolytic processing should happen, releasing the individual peptides. However, only three MBPs (MBP2, MBP3 and MBP4) showed positive antimicrobial activity predicted *in silico* by CS-AMPPred (Supplementary Table S2). At the end of screening, 17 uncharacterized AMPs (3 defensins, 3 snakins, 8 lipid transfer proteins and 3 α-harpinins) were obtained (Supplementary Fig. S5A-D).

**Figure 3:**
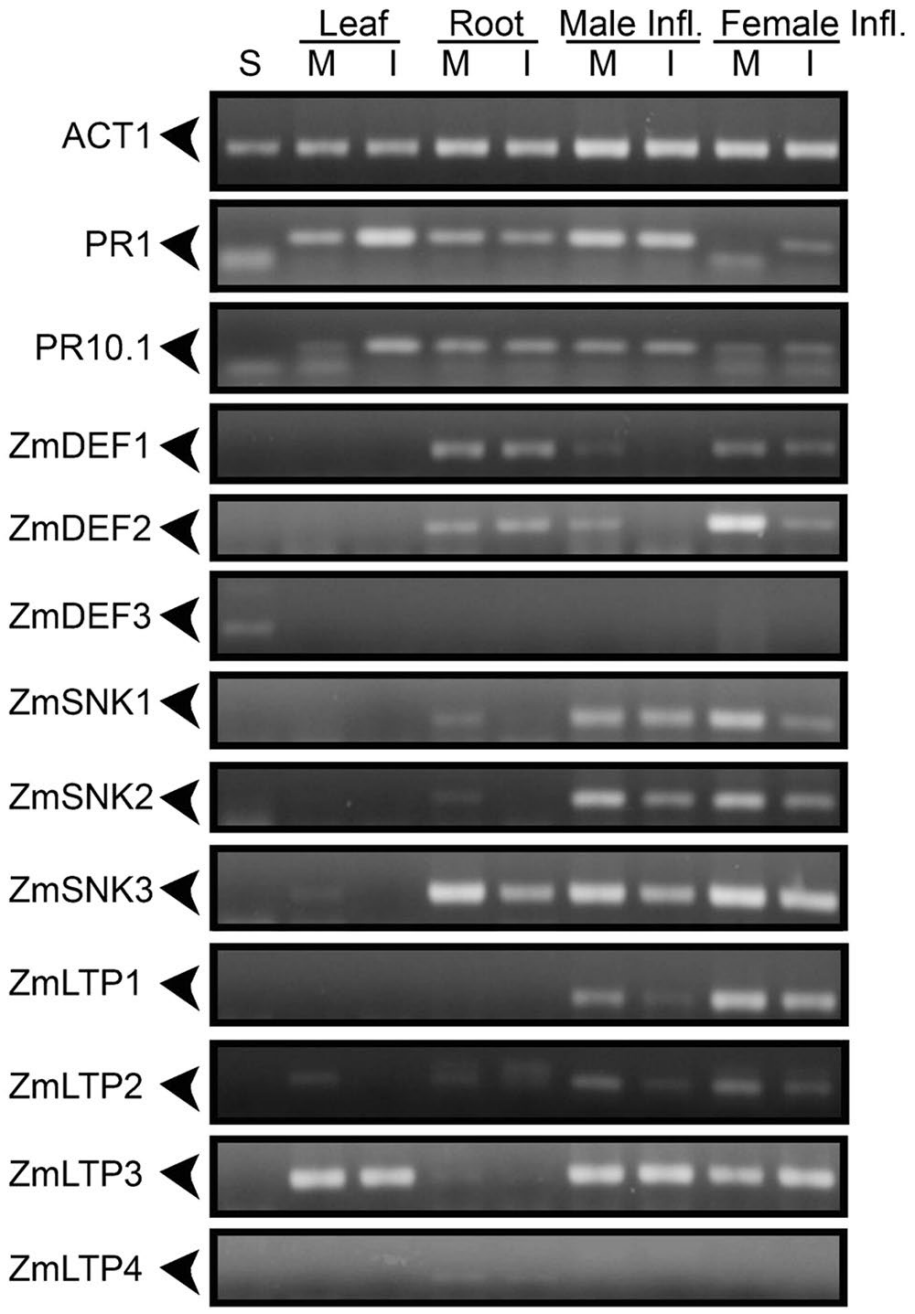
Semi-quantitative RT-PCR of uncharacterized AMPs from NCBI database. S - seeds; M - mock-inoculated; I - inoculated; Male Infl. - male inflorescence; Female Infl. - female inflorescence. Visualization by 2% agarose gel stained with ethidium bromide. The picture represents results of three independent biological replicates.

Semi-quantitative RT-PCR (sqRT-PCR) was carried out with these uncharacterized AMPs, aiming to validate the gene expression in different organs of maize and in response to *C. graminicola* infection (Fig. 3). The MBP precursor was excluded from this validation since it is already described with expression in seeds^21^. From 16 novel AMPs, only four LTPs (*ZmLTP5, ZmLTP6, ZmLTP7* and *ZmLTP8*) did not have their expression detected in any of the examined organs, and these were discarded from the screening. Two stress marker genes (*PR1* and *PR10.1*) were included in this validation in order to verify local and systemic defense induction against *C. graminicola*. Gene expression induction of *PR1* and *PR10.1* gene markers was found in infected leaves and female inflorescence SAR^+^ (Fig. 3). However, no modification in the gene expression of these markers was detected in infected root and male inflorescence SAR^+^. Within the defensins group, two defensins (*ZmDEF1* and *ZmDEF2*) are expressed in the root and showed a small down-regulation in both inflorescences SAR^+^ after infection (Fig. 3). The third defensin was specifically expressed in seeds. In the snakin group, all three *ZmSNKs* are expressed in roots and inflorescences. A down-regulation of *ZmSNK1* and *ZmSNK2* was observed in roots infected with *C. graminicola*. Within the LTP group, a diverse expression pattern was observed for members of this class, including constitutive expression (*ZmLTP2* and *ZmLTP3*), root-specific expression (*ZmLTP4*) and male and female inflorescence expression (*ZmLTP1*). From this validation we confirm the existence of 9 novel AMPs with mis-annotation from the NCBI database and observed that most of the analyzed AMP classes are expressed in inflorescences and root organs. However, five of these AMPs were not regulated by *C. graminicola* infection, and down-regulated *ZmSNKs* may be related to a low concentration of gibberellic acid (GA_3_), the hormone necessary for transcription induction.

### RNA-Seq analyses

**Figure 4:**
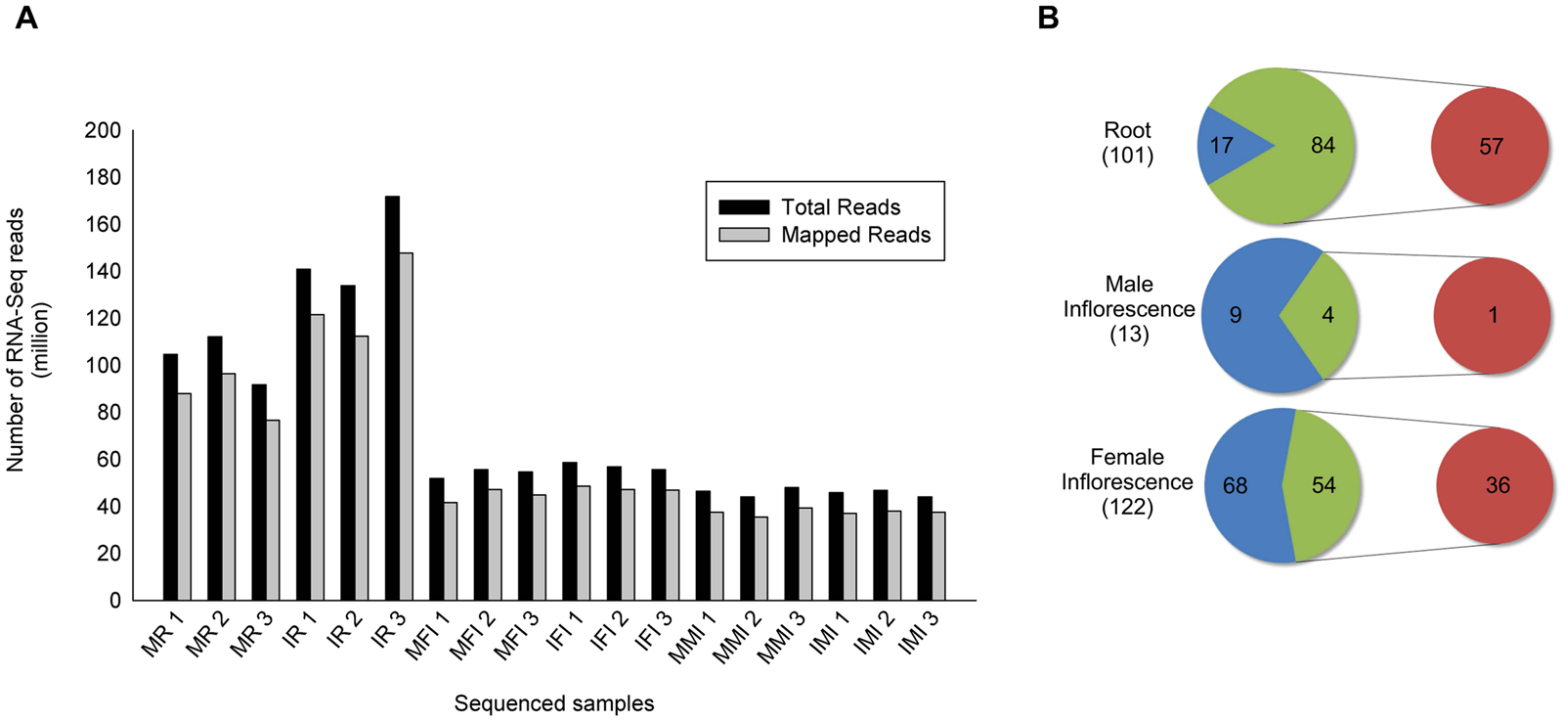
Summary of sequence analysis. (**A**) Mapping statistics of RNA-Seq data. Black bars represent the total numbers of sequenced reads for each sample, and grey bars represent reads mapped in the maize genome AGPv3.23. Abbreviations: Mock Root, MR; Inoculated Root, IR; Mock Female Inflorescence, MFI; Inoculated Female Inflorescence, IFI; Mock Male Inflorescence, MMI; Inoculated Male Inflorescence, IMI, with their respective biological triplicate. (**B**) DEGs obtained from DESeq2. Blue, annotated genes; green, uncharacterized genes; red, functionally re-annotated genes (InterProScan).

Aiming to study local and systemic acquired resistance in maize in response to the fungus *C. graminicola*, an Illumina RNA-Seq approach was performed. Root locally inoculated (7 d.a.i) with *C. graminicola* and male and female inflorescences (12 d.a.i) of *C. graminicola*-inoculated plants in leaves and roots were collected in biological triplicate. The same RNA used in AMP validation was used for transcriptomic analysis. However, only root, male and female inflorescence organs were sequenced due to the presence of gene transcripts related to major classes of AMPs (Supplementary Fig. S6). cDNA sequencing for root samples yielded an average 125,882,730 reads per sample. Male inflorescences showed a yield of 55,581,817 reads, and in female inflorescences the yield averaged 45,872,914 reads for each sample (Fig. 4A). A greater sequencing depth was requested in roots to retrieve fungal genes. However, a low depth of reads mapped against the *C. graminicola* genome was obtained. These fungal sequences were completely excluded from analysis. The result of sequencing for all samples presented reliable quality, with 87.3–92.4% of base call higher than Q_30_ (Supplementary Table S3). The overall alignment rate in the maize genome was on average 83.2% for all samples, indicating genetic compatibility between BRS1010 and B73 varieties, and aligning rate for fungus was less than 1% (Fig. 4A).

### Local defense response in root infected by *Colletotrichum graminicola* using RNA-Seq

The differential expression analyses in root between control and infected samples were performed using scripts from the DESeq2 package, showing 101 genes were differentially expressed. Among them, 84 genes were annotated as uncharacterized genes (Supplementary Table S5). Among uncharacterized genes, 57 genes were functionally re-annotated based on the domain signature by InterProScan (Fig. 4B and Supplementary Table S6).

**Figure 5:**
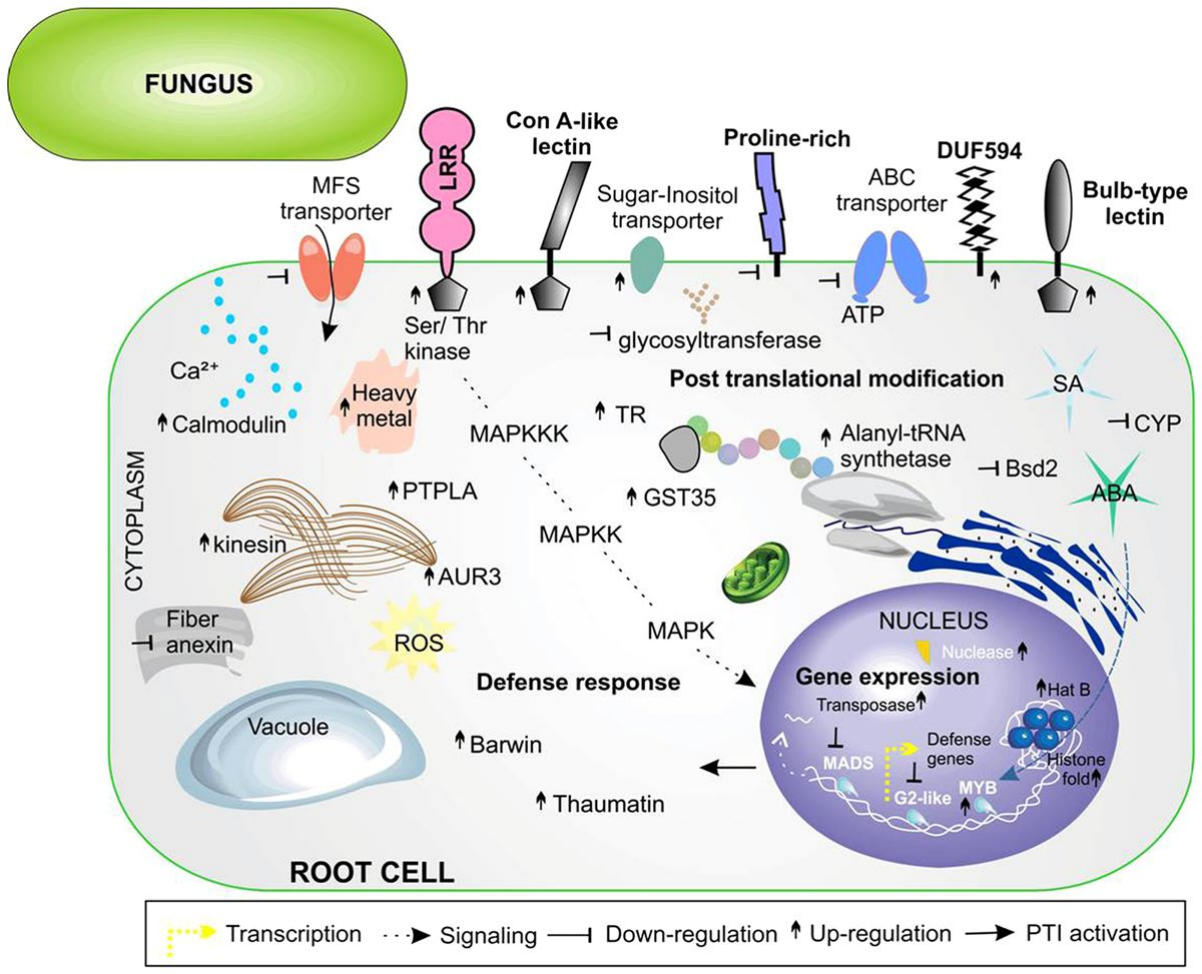
Maize root cell showing the predicted location of DEGs. Root localized acquired resistance was activated against *C. graminicola* infection. Predicted locations in the cell of proteins encoded by DEGs were obtained by gene ontology (GO) annotation and domain signature (InterPro). Symbols near the name of genes indicate gene regulation. Fold change values of each gene are in Supplementary Table S5.

Most differentially expressed genes (DEGs) in the root involve integral membrane components. Here, induced gene expression was found for leucine-rich repeats receptor (LRR), classic sensors to detect PAMPs, in response to *C. graminicola* infection (Fig. 5). The fungal effectors’ recognition seems not to be related to proline-rich receptor, since that was down-regulated in inoculated roots. Among the uncharacterized DEGs checked by domain were several lectin receptor kinases (LecRKs) indicating a possible role in *C. graminicola* recognition (Table 1). Induction of expression of a gene encoding a transmembrane protein with a DUF594 domain was also found, and this can be related to fungal recognition. DEGs encoding transporters cell wall-anchored, major facilitator superfamily (MFS) and ABC transporters were down-regulated, while a gene encoding sugar-inositol transporter was up-regulated (Fig. 5).

**Table 1:** Types of receptor-like kinases, differentially expressed from root and systemic inflorescences, in response to *C. graminicola* infection.

RLK type	Organ*	Gene ID	Regulation^1^	Motifs	InterPro
LecRKs	R	GRMZM2G168985/GRMZM2G454511	↓	Bulb-type lectin domain	IPR001480
LecRKs	R	AC218998.2_FG007	↑	Jacalin-like lectin domain	IPR001229
LecRKs	R	GRMZM2G487328/GRMZM2G034611/GRMZM2G079219/GRMZM2G113421/GRMZM2G172386	↑	Concanavalin A-like Lectin/glucanase domain	IPR013320
DUF295	R	GRMZM5G823824	↓	Domain of unknown function DUF295	IPR005174
DUF594	R	GRMZM2G032551	↑	Domain of unknown function DUF594	IPR007658
WAK	R	GRMZM2G093072/GRMZM2G172386/GRMZM2G333045	↑	Wall-associated receptor kinase, galacturonan-binding domain	IPR025287/IPR032872
LRR	R	GRMZM2G151738/GRMZM2G048801/GRMZM2G029211	↑/↑/↓	Leucine-rich repeat-containing N-terminal, plant-type	IPR032675/IPR013210
PERK	MI	GRMZM2G021289	↓	Proline-rich extensin-like	PR01217
LysM	FI	GRMZM2G008773	↓	LysM domain	IPR018392
FAS1	FI	GRMZM2G021794	↑	FAS1 domain	IPR000782
LecRKs	FI	GRMZM2G448672	↑	Concanavalin A-like Lectin/glucanase domain	IPR013320
LRR	FI	GRMZM2G016477/GRMZM2G162829/GRMZM2G016477/GRMZM2G119490	↓/↓/↓/↑	Leucine-rich repeat-containing N-terminal, plant-type	IPR032675/IPR003591/IPR013210
DUF761	FI	GRMZM2G162396/GRMZM2G395983	↑/↓	Protein of unknown function DUF761	IPR008480
DUF1475	FI	GRMZM2G092256	↑	Protein of unknown function DUF1475	IPR009943
PERK	FI	GRMZM2G099802	↓	Proline-rich like	IPR006041

*R, root; MI, male inflorescence; FI, female inflorescence. ^1^↓down-regulated and ↑ up-regulated in control samples versus inoculated (individual fold change values are in Supplementary Tables S5–S10).

Receptor kinase anchored to the membrane can initiate a cascade signaling involving phosphorylation and glycosylation. Two DEGs (GRMZM2G181266 and GRMZM2G301389) encoding protein-tyrosine phosphatase-like enzymes (PTPLA) were up-regulated and were predicted to be involved in post-translational modification, which can create novel recognition motifs for protein interactions, affecting protein stability. Three DEGs (GRMZM2G037617, GRMZM2G107645, GRMZM2G337109) encoding serine/threonine-protein kinase aurora-3 (AUR3) were up-regulated and may be related to cell division program. These events are consistent with increased expression of a DEG encoding kinesin-like protein associated with microtubules (Fig. 5). Two other genes (GRMZM2G102015, GRMZM2G063798) involved in signaling proteins were also up-regulated: a dephospho-CoA kinase, the enzyme that catalyzes the final step in CoA biosynthesis and CDP-alcohol phosphatidyltransferase (CDIPT), involved in phospholipid biosynthesis (Supplementary Table S6). Besides phosphorylation, another signaling process in plant defense response consists of the transfer of sugars to acceptor molecules^22^. A decrease was observed in glycosyltransferase gene expression, with three members of this family down-regulated and only one member up-regulated. Calcium signaling through the cell membrane may be happening due to the induction of calmodulin gene expression. A maize cell response after *C. graminicola* infection is the production of ROS by changing the cellular redox environment. Two DEGs encoding proteins involved in cellular redox homeostasis maintenance were activated, a glutathione-S transferase (GST35) and thioredoxin (TR) (Supplementary Table S6). Induction of gene expression of an alanyl-tRNA synthetase was verified.

Another predicted cell compartment that contains a large number of proteins encoded by DEGs is the nucleus. Two DEGs (GRMZM2G480621, GRMZM5G809663) encoding proteins with histone fold domains were annotated between uncharacterized genes. In addition, a DEG encoding histone acetyltransferase type B (HAT B) was found up-regulated in *C. graminicola*-inoculated roots, indicating a possible histone acetylation. The DEG encoding a ZmMYB transcription factor (TF) involved in response to ABA was up-regulated, indicating this hormone as a marker of local fungal infection. In contrast, DEGs encoding ZmMADS and ZmG2-like TFs were down-regulated (Fig. 5). These TFs are involved in DNA-binding and dimerization. Four DEGs members of the Ptta/En/Spm transposase family were activated in response to infection (Supplementary Table S6). In parallel with this event gene expression was induced of two DEGs (GRMZM2G005536 and GRMZM2G148626) encoding DNA polymerase subunits, Cdc27.

**Figure 6:**
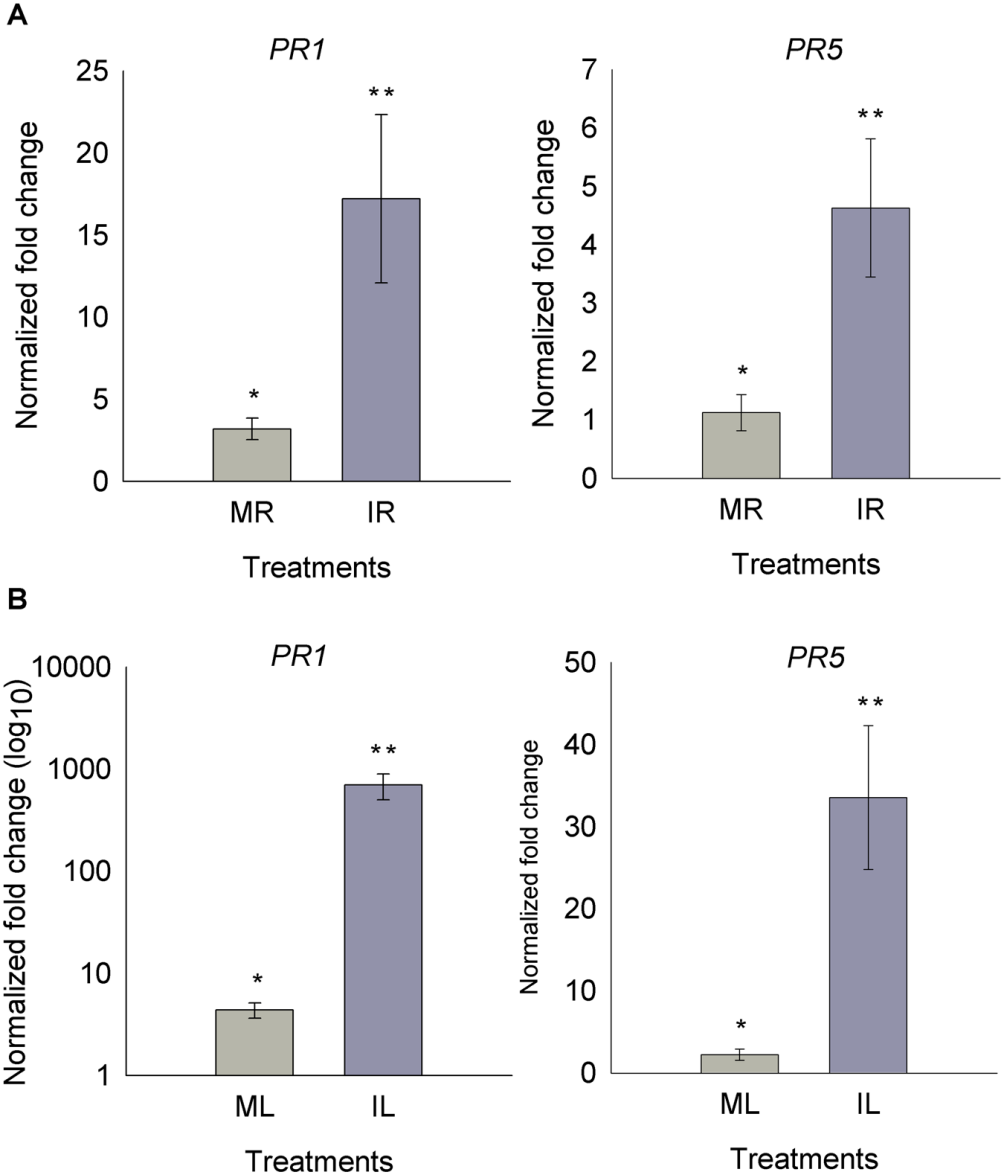
PR1 and PR5 transcript level quantified by Real time PCR in maize plants. Light gray, PR transcripts quantification in mock-root (MR) and inoculated-roots (IR) with *C. graminicola*, dark grey, (**A**). PR transcripts quantification in mock-leaf (ML) and inoculated-leaf (IL) with *C. graminicola*, (**B**). Genes were normalized to glyceraldehyde 3-phosphate dehydrogenase (GAPC). Values indicated are the mean (±standard error) of three independent biological replicates. T-test was used to show statistical difference. Root - PR1, p-value 0.0434; PR5, p-value 0.0170. Leaf - PR1, p-value 0.0295; PR5, p-value 0.0067.

In addition to cell surface signaling there is intracellular signaling with the accumulation of defense-related proteins. As reported for leaf-*C. graminicola* interaction, infected root activates thaumatin (PR5) gene expression in response to *C. graminicola*. Here, real-time PCR was used to confirm the transcript accumulation of stress marker genes in leaf and root locally inoculated (LAR) with *C. graminicola*. It was found that inoculated leaves increased expression of PR1 and PR5 around 600 and 30 times, respectively, compared to mock leaves. However, the slighter increase of 17 and 5 times was observed in inoculated roots when compared to negative control (Fig. 6). Among uncharacterized DEGs, a gene encoding a defense protein containing Barwin domain with signal peptide was up-regulated.

### Gene expression associated with systemic acquired resistance in inflorescences of maize inoculated with *Colletotrichum graminicola*

Differential gene expression analysis indicated that the activation of defense was more strongly observed in female inflorescence than male inflorescence. In male inflorescence 13 DEGs were found between mock and inoculated samples; from these, four are hypothetical proteins (Fig. 4B and Supplementary Table S7). In female inflorescence, 122 genes were differentially expressed. Of this total, 54 DEGs were annotated as hypothetical proteins (Supplementary Table S9). For both inflorescences the hypothetical proteins were functionally re-annotated based on the signature of domain by InterProScan (Fig. 4B and Supplementary Tables S8 and S10).

**Figure 7:**
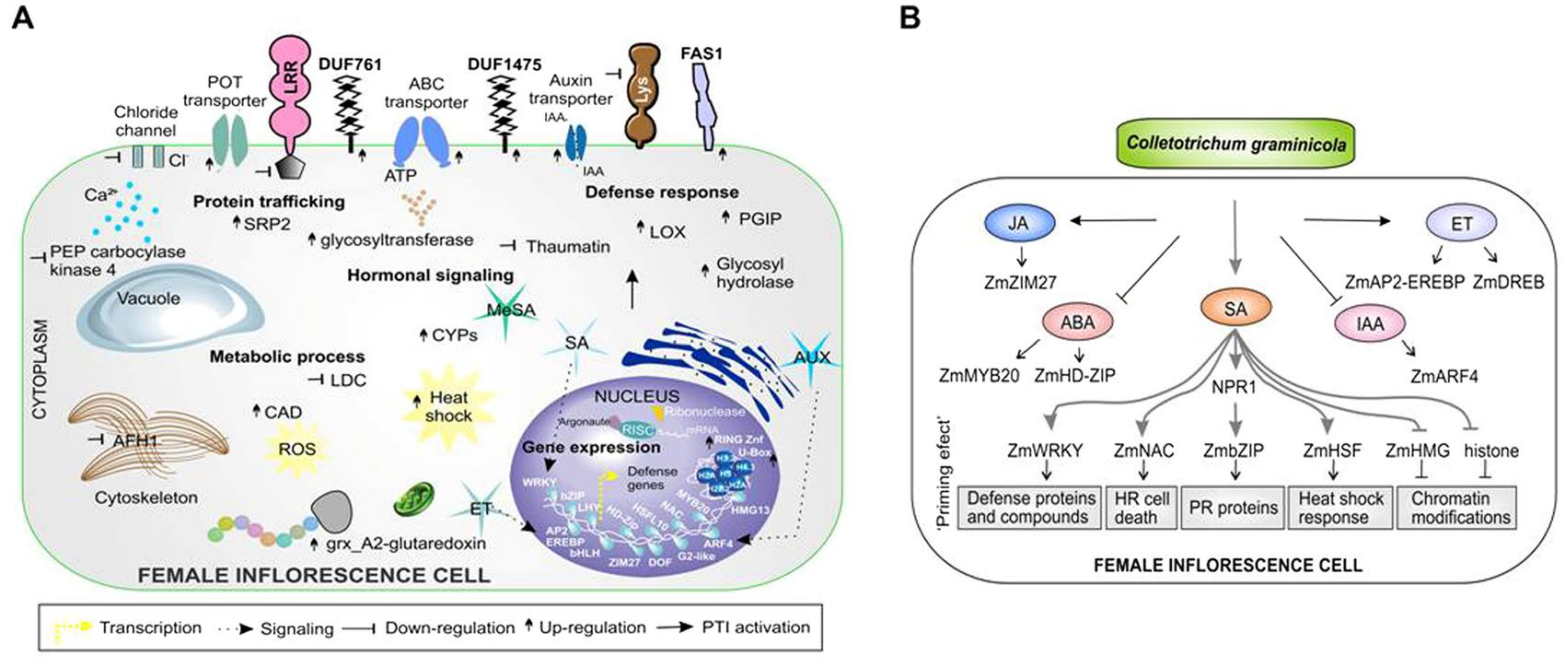
SAR signaling in maize inflorescence based on DEG analysis. (**A**) Maize female inflorescence cell showing the predicted location of DEGs between SAR^+^ and SAR^−^ plants. Fold change values of each gene are in Supplementary Table S9. (**B**) Hormone signaling crosstalk in systemic female inflorescence inoculated with *C. graminicola*. SA-dependent signaling pathway involves changes in redox environment, leading NON-EXPRESSOR OF PR1 (NPR1) to move from cytoplasm to the nucleus. NPR1 can interact with ZmbZIP TF, which induces transcription of PR proteins. Other positive regulators in SA signaling pathway are ZmWRKY, ZmNAC, ZmHSF and ZmHMG responsible for the priming effect induction. Chromatin modifications seem to be happening due to a decrease in gene expression of ZmHMG TF related to disassembly of nucleosome and by decrease in gene expression of histones. Signaling pathways dependent on auxin and ABA are not involved in SAR activation in inflorescences. JA/ET-dependent signaling pathways appear to contribute to systemic defense against *C. graminicola*.

In male and female inflorescences, a decrease in the gene expression encoding proline-rich extensin-like receptors (PERK) was observed, indicating that systemic signal perception cannot occur in this pathway. Similarly, in female inflorescence a decrease in the gene expression of LRR receptors and receptor with Lys domain was observed (Table 1). However, the increase in the expression of genes encoding a membrane protein with FAS1 domain (cell adhesion domain), one LecRK, two membrane proteins with DUF761 and DUF1475 motifs were observed (Supplementary Table S10). Also in relation to cell membrane proteins, a DEG encoding the chloride channel had a decreased expression. In contrast, an increased gene expression of ABC transporters and a proton-dependent oligopeptide transporter (POT) were verified (Fig. 7A).

Regard hormonal changes, in female inflorescence SAR^+^ the induction of gene expression of an auxin efflux carrier in the cell membrane was observed. Similarly, MeSA seems to be involved in systemic defense in this organ due to the gene expression induction of a carboxyl methyltransferase involved in its biosynthesis. Five genes, members of the cytochrome P450 family (CYP), had their expression induced in female inflorescence (Supplementary Table S9). In male inflorescence, systemic intracellular signaling involves the induction of gene expression of an endo-1,4-beta-mannosidase and a glycosyltransferase from family 8. A glycosyltransferase gene was also found to be up-regulated in female inflorescence SAR^+^. Modifications in metabolic processes such as phytoalexin biosynthesis were suggested by repression of a gene encoding a N-hydroxycinnamoyl/benzoyl transferase. In female inflorescence, a putative cinnamyl-alcohol dehydrogenase family gene (CAD), involved in biosynthesis of phenylpropanoids, was up-regulated (Fig. 7A). One gene encoding lysine decarboxylase-like (LDC) protein which catalyzes the first step in the biosynthetic pathway of quinolizidine alkaloids was down-regulated. Similar to defense response locally activated in the root after *C. graminicola* infection, changes in cellular redox state were observed in female inflorescence SAR^+^. Three genes (GRMZM2G441906, GRMZM2G311898, GRMZM2G063896) of the glutaredoxin family involved in keeping the cellular redox homeostasis were up-regulated in response to fungal infection. A gene encoding heat shock protein was also up-regulated in this organ. Beyond these events, modification was observed in the cytoskeleton by down-regulation of a formin (AFH1) gene involved in actin polymerization, and modifications in the process involved in protein translation verified by the induction of an amidase gene and a signal recognition particle protein (SRP2).

Interestingly, most DEGs in the female inflorescence SAR^+^ encode proteins predicted to be located inside the nucleus. Ten gene-encoding histones, including members of the H3.2, H2A1, H4.3, H2A, H3, H2B2 and H4 subfamilies, showed a decreased gene expression (Supplementary Tables S9 and S10). According to this event, the decreased expression of a ZmHMG13 TF is involved in the mounting of nucleoprotein complexes. The pathway of protein degradation by ubiquitin proteasome system (UPS) seems to be required for systemic defense activation. DEGs encoding a putative RING zinc finger protein and a protein containing U-box domain, both E3 ubiquitin ligases, had their expression induced in female inflorescence SAR^+^. Induced gene expression was also verified of two proteins containing F-box domain involved in protein-protein interaction and formation of complexes with E3 ligase.

Changes in gene expression of TFs were more strongly observed in female inflorescence than male inflorescence. DEGs encoding ZmAP2-EREBP TFs and dehydration-responsive element-binding protein (DREB), both involved in the ethylene signaling hormone, were up-regulated in male and female inflorescence SAR^+^. This event is consistent with the increase in senescence rate observed in these organs. A transposon was also found up-regulated in this organ. In female inflorescence, a DEG encoding a protein containing the GATA domain present in TFs that function as transcriptional activators was up-regulated. This same organ showed DEGs related to TFs involved in hormonal changes. The *ZmARF4* gene was down-regulated, and this TF binds to auxin response elements^23^. The *ZmZIM27* gene was up-regulated and this TF binds to jasmonic acid response elements^24^. The gene expression induction of a ZmWRKY TF involved in the signaling of SA hormone and related to defense activation was observed in female inflorescence SAR^+^ (Fig. 7B). Also, the *ZmbZIP* gene was up-regulated and belongs to the TGA TF family, regulating SA-responsive genes. This seems to be the main pathway of SAR activation in female inflorescences. Downstream of defense-related genes is induction of the gene expression of ZmNAC and ZmHSF TFs, both involved in plant cell death and hypersensitive reaction (Fig. 7B). In addition to previously described TFs, other DEGs encoding ZmHD-Zip, ZmbHLH134, ZmMYB20 and ZmDOF were observed to be down-regulated.

No gene encoding PR proteins was differentially expressed in male inflorescence SAR^+^ in response to *C. graminicola*. However, in female inflorescences SAR^+^, induction of gene expression of a polygalacturonase inhibitor 1 (PGIP) was observed. A gene encoding the enzyme lipoxygenase (LOX), which catalyzes the dioxygenation of polyunsaturated fatty acids in lipids, was also up-regulated in this organ. Contrary to what is observed in local root infection by *C. graminicola*, a decrease in thaumatin gene expression was observed in female inflorescence SAR^+^. No statistically significant differences in gene expression were observed for AMPs in local and systemic infections of maize in response to *C. graminicola*.

## Discussion

The pathosystem of maize infected by *C. graminicola* has been an appropriate comparative model to understand plant defense mechanisms against biotrophic and necrotrophic fungi in the same pathogen. Previous works have studied LAR in root and leaves after *C. graminicola* infection^2, 6^. However, these works did not use an accurate high-throughput approach to look for DEGs involved in defense, including genes with low abundance transcripts, such as AMPs. In this work we studied SAR in inflorescences because reproductive organs are good sources of defense compounds, including the gene expression of AMPs. The potential for floral tissues as a source of defense molecules was observed in the protein extract of *Zantedeschia aethiopica* in which they showed high inhibition of *E. coli* growth^25^. Silverstein^26^ also showed that several classes of AMPs are expressed in reproductive tissues of plants^26^. Here, we used a recent screening strategy using sequence motif models (cysteine patterns) to search for novel AMPs from the NCBI database, aiming to explore public information. Many putative representatives of traditional classes of AMPs were found without annotation and with expression mainly in reproductive organs. However, most of the analyzed uncharacterized AMPs showed no variation in gene expression in response to *C. graminicola* infection in sqRT-PCR analysis. Likewise, there was no change in gene expression of AMPs within the transcriptome. These results suggest that since maize has a large spectrum of AMPs, the *C. graminicola* infection does not regulate the accumulation of AMP transcripts in compatible interactions. However, the novel AMPs found in NCBI’s screening can be isolated from maize and experimentally characterized for their antimicrobial activity in future experiments.

Expanding the analysis to transcriptomic level it was found that, even in the absence of anthracnose symptoms in root, there is evidence of local activation of PTI against *C. graminicola*. Among the cellular components that are locally highly modified in the root are integral membrane proteins and proteins inside the nucleus. Protein containing a Leu-rich region (LRR) was previously reported as important for *C. graminicola* recognition on maize leaves, when starting the activation of defense^6^. Furthermore, novel LecRKs were found up-regulated, indicating a central role in *C. graminicola* recognition. Members of this superfamily with glucanase and chitinase activity may change the integrity of hyphal walls, giving rise to elicitor chitin and β-1,3-glucan fragments, which can trigger maize immunity. It is well known that β-1,3-glucan of *C. graminicola* is required for cell wall rigidity in appressoria and for fast-growing necrotrophic hyphae^27^. However, this glucan can be an easy target for plant surface receptors. WAK and DUF-type receptors were also activated in root inoculated with *C. graminicola* and are promising targets to be validated with gene knockout/overexpression strategies. Previous studies reported that WAK receptor expression is required during a response to the pathogen, because their induction is necessary for plants to survive high levels of salicylic acid^28, 29^. A DUF26 receptor type was also found induced by oxidative stress and SA treatment in *Arabidopsis thaliana* ^30^. Many signaling pathways related to pathogen recognition, like MAPK signaling cascades, calcium-dependent signaling, glycosyltransferases and oxidative stress were observed in root. Primarily, hormones orchestrate these signals until reaching the nucleus. Previous work has shown the involvement of ABA and SA in the induction of local defense response to *C. graminicola* in leaf of maize^6^. It was also reported that root of maize infected by *C. graminicola* increases levels of concentration of SA, JA and ABA to high when compared to leaf. In addition, root and leaves locally infected increased accumulation of SA and ABA in systemic leaves, inducing high systemic resistance against secondary *C. graminicola* infections^2^. Systemic acquired resistance has been orchestrated by many chemical inducers, such as SA, JA, methylated derivative MeSA and auxin^31^. Here we find that root inoculated with *C. graminicola* also activates SA-inducible defense genes in reproductive organs, in agreement with the previously published works. This is important to ensure harvest crops with little contamination with *C. graminicola*. In addition, we noted the involvement of DEGs related to ET, JA and MeSA hormones, which may be involved with late infection by *C. graminicola*. Senescence and programmed cell death (PCD), regulated by ABA and ethylene hormones (ET), are mechanisms used by plants to try to stop the progress of pathogenic infection. The increase in senescence was stronger in male inflorescence than female inflorescence (Fig. 1C and D).

Interestingly, we found that the greatest changes in female inflorescence SAR^+^ involve a massive reprogramming of gene expression with increased ZmWRKY TF, traditionally involved in SAR activation, and increased expression of other TFs such as ZmbZIP, ZmNAC and ZmHSF, also involved in plant defense. Previous studies have reported that SAR induction requires SA accumulation, which induces promoters of PR1, WRKY6 and WRKY53 genes^17^. These TF modifications were related to the remodeling of chromatin through decreased expression of histones and ZmHMG13 TF involved in mounting nucleosome complexes. The decrease in histone gene expression indicates that a possible nucleosome disassembly is required for DNA unfolding to improve DNA access to transcription factors (TFs) involved in defense activation. Chromatin states control cellular memory and differentiation in animals and plants^32^. Recent works have shown that priming of innate immunity is correlated with chromatin modification by methylation/acetylation and ubiquitination of histones^33^. Here, we found that histone acetylation may be required for induction of LAR at the root. In addition, SAR signaling with chromatin modifications can be passed to the next generation of plants, which will provide a rapid activation of defense-related genes in secondary infections. This transgenerational resistance has been observed in Arabidopsis, in which progeny from *P. syringae*-inoculated Arabidopsis (P1) were primed to activate SA-inducible defense genes and were more resistant to the hemibiotrophic pathogens *Hyaloperonospora arabidopsidis* and Pst DC3000 when compared with progeny from control-treated Arabidopsis. Transgenerational SAR involving chromatin remodeling requires PR1 protein and is associated with priming of SA-dependent defense^17^. Here, the *PR1* gene was found to be up-regulated in leaf and root in response to *C. graminicola* (Fig. 6) and in female inflorescence SAR^+^ (Fig. 3), indicating that a similar signaling pathway of transgenerational SAR of Arabidopsis may be happening in maize.

The primed state induced in female inflorescences involves many local defense responses, such as induction of genes related to oxidative stress, glycosyltransferase and increased expression of defense-related genes like LOX and PGIP. This PGIP inhibitor seems to be involved in antifungal response, since it inhibits enzymes that are capable of degrading pectin from plant cell walls (fungal polygalacturonase). The enzyme lipoxygenase (LOX), which catalyzes the dioxygenation of polyunsaturated fatty acids in lipids, has been widely reported to be involved in pathogen attack signaling, and previous work has shown its involvement in systemic resistance against bacteria^34^. Another agent that may be operating in root defense is Barwin-like protein, induced and secreted by the extracellular surface. Among the systemic defenses observed in female inflorescence is the induction of gene expression of enzymes that participate in the production of secondary metabolites (phytoalexins and phenylpropanoids), indicating that chemical defense is also acting. Balmer^2^ reported that high amounts of flavonoids such as narigenin chalcone, apigenin and genkwanin are important for local defense in root and leaf against *C. graminicola*. The authors also found that apigenin, genkwanin and chlorogenic acid led to a dose-dependent reduction of radial growth of *C. graminicola*, indicating that maize uses a chemical arsenal to contain the infection by *C. graminicola*.

## Conclusions

This article shows that the main agents that appear to act in maize defense are polygalacturonase inhibitor 1 (PGIP), Barwin proteins, PR proteins and secondary metabolites. We have demonstrated that *C. graminicola* induces SAR in inflorescences by a SA-dependent signalization, but JA/ET signaling pathways also contribute to systemic defense. Gene expression induction of ZmWRKY, ZmbZIP, ZmNAC and ZmHSF TFs and chromatin modifications may be essential for priming effect induction in female inflorescences SAR^+^ against *C. graminicola*. In *C. graminicola-*inoculated roots, SA and ABA hormones appear to be necessary to induce the defense. LecRKs present in the cell membrane play a fundamental role in *C. graminicola* recognition and root LAR activation. Together these results indicate essential components in signaling defense against *C. graminicola*. Strategies involving over-expression, gene knockout or signal-transduction master switches of key components of defense signaling may be used in genetic engineering of resistance against *C. graminicola* in maize^20^.

## Methods

### Plant Material and growth conditions

Seeds of *Zea mays* (accession BRS1010, with high productivity, resistance to pathogens and good adaptation to Brazil’s climate), susceptible to *C. graminicola*, were obtained from Embrapa Maize and Sorghum, (EMBRAPA/CNPMS - MG, Brazil). Seeds were surface-sterilized by soaking in 500 ml of solution of 2% sodium hypochlorite under strong agitation for 5 min. Seeds were then washed three times in sterile water, finally being used for seeding in sterile soil. Some seeds were frozen in N_2_ liquid for RNA extraction. Plants were grown in a greenhouse with natural photoperiod, temperature 19–32 °C and high humidity. Plants were inoculated at V4 stage of development for NCBI screening validation. To perform the microscopy, humid chambers were built with sterile plastic bags for each potted plant, to increase the humidity.

### Fungal material and growth conditions

*C. graminicola* strain 03.10 M was obtained from the mycology collection of Embrapa Maize and Sorghum. Fungal growth was carried out in Petri dishes containing medium oatmeal agar culture (FAA) and kept at ambient temperature (25 °C) under continuous fluorescent light. After three days of growth, there was scraping of the mycelia to induce sporulation of fungus. After 14–21 days of growth, water was added to the culture medium. Through surface scraping of mycelia, conidia were isolated, and this suspension (water + spores) was filtered into previously sterilized gauze and conidia collected in a plastic tube. After achieving a conidial suspension of the desired concentration for inoculation of plants, the solution was washed three times by adjusting the volume to 50 mL by adding sterile water. A step of centrifugation at 3000 g for 3 min took place, to pellet conidia. After the last wash, spore concentration was adjusted to 10^6^ conidia ml^−1^ with sterile water and by addition of 0.01% of Tween20.

### Plant fungal infection

Maize roots at V4 stage were inoculated with 100 ml of spore suspension (10^6^ conidia. ml^−1^). Three holes, close to main plant root (around 3 cm), were made to facilitate the spread of spores. 0.01% Tween-20 was used for the control plants’ inoculation. Maize leaves at V4 stage were placed in a horizontal position, and each of the three leaves of the plant were sprayed with spore fungal solution until point of runoff. Then, plants were again placed in a vertical position. Water and Tween-20 was used for inoculation of control plants. These samples were used in sqRT-PCR analysis. In inflorescences SAR^+^, plants were grown until the beginning of flowering (VT-R1 stage) and then were inoculated on leaves (by spraying) and on roots (addition of 100 ml of spore solution in soil), simultaneously, and, after observation of early symptoms in inoculated leaves, the inflorescences were collected. Three biological replicates were collected for each treatment and each replicate consists of individual plants.

### Microscopy analyses

Light microscopy was performed using an *Axiophot* microscope (Zeiss) to check the correct progress of Anthracnose disease. Control-inoculated leaf discs were collected 24, 36, 48 and 72 h.a.i. Inoculated and control roots-were collected 48 h.a.i. Three biological replicates were collected for each sample. In order to observe early fungal infection events, fragments of leaves (~50 mm) were fixed in FAA (formaldehyde: acetic acid:70% ethanol in 1:1:18 proportion) for 24 h at room temperature and then kept in 70% ethanol at 4 °C. Leaves were bleached using acetic acid and stained with lactophenol cotton blue (100 ml lactophenol, 1 ml of aqueous 1% cotton blue, 20 ml of glacial acetic acid) and visualized by light microscopy^35^. Events during fungal colonization were evaluated in samples of leaves 36 and 48 h.a.i fixed in 0.1 M cacodylate buffer solution, pH 7.2 containing 2.5% glutaraldehyde and 2% formaldehyde for 24 h at 4 °C. Samples were washed in cacodylate buffer and then fixed in cacodylate buffer containing 0.5% osmium tetroxide in the dark for two 1 h periods at room temperature. Subsequently, samples were washed with buffer and distilled water and dehydrated in solutions with increasing ethanol concentrations (30%, 50%, 70%, 90%–30 min each and 100% for three times of 20 min). After this step, the samples were gradually infiltrated with Epon resin inclusions and polymerized at 72 °C for 48 h^36^. Sections containing 3 μm were obtained and stained with toluidine blue (0.1 g borax and 0.1 g toluidine O in 100 ml distilled water) and observed under light microscopy.

### Sample preparation and RNA extraction

Roots, leaves, male and female inflorescence (control-inoculated for all) and seeds were collected, immediately frozen in liquid N_2_ and used for RNA extraction. RNA was extracted using Trizol reagent (Invitrogen). Quality and RNA integrity were confirmed by 1% agarose gel staining with ethidium bromide and by Agilent 2100 Bioanalyzer^37^. All RNAs were quantified using Quant-iT-^TM^ RNA Assay Kit^38^.

### Screening for uncharacterized classes of AMPs in NCBI database

Cysteine patterns described by Silverstein^26^ for the main AMP classes^26, 39^ were used for AMP identification in the NCBI RefSeq proteins annotated for maize variety Japonica (Supplementary Table S1). The search performed through PERL script was refined to select uncharacterized sequences with fewer than 350 amino acid residues. The presence of a signal peptide and transmembrane domains was predicted by Phobius^40^. Sequences obtained confirmed the annotation in InterProScan^41^, considering the larger domain for the annotation. The antimicrobial activity was predicted by CS-AMPPred^42^. Retrieval of the α-harpinin class was carried out by local sequence alignment (NCBI BLASTP against the same database, using the public MBP-1 peptide as query sequence.

### Gene expression validation of uncharacterized AMPs by Semi-quantitative Reverse Transcriptase PCR

To validate the gene expression of potential novel AMPs found in the NCBI database, primers were designed for each UniGene using the Primer 3 program, and the specificity of amplification was checked using the PrimerBLAST tool of NCBI and alignment against the maize genome (Supplementary Table S4). Parameters used to design primers were 18–20 bp in size, 60–62 °C of annealing temperature and 50% of GC content. Primers were also designed for an actin constitutive gene to normalize RNA quantity in different organs. cDNA was synthesized from 1 ug of total RNA extracted from each organ. cDNA synthesis was performed using the kit SuperScriptTM III First-Strand Synthesis SuperMix for qRT-PCR (Invitrogen). All RNA was treated with DNase I (Kit DNaseI Amplification Grade – Invitrogen). To verify AMP gene expression, PCR reactions were conducted with 20X diluted cDNA, 1X reaction buffer, 200 nM of each oligonucleotide, 250 uM dNTPs, 2.5 mM MgCl2 and 2.5 U of recombinant Taq polymerase (Invitrogen). The PCR program used was an initial step of 95 °C for 3 minutes, followed by 35 cycles of 95 °C for 10 seconds, 55 °C for 10 seconds and 72 °C for 15 seconds, finishing with 72 °C for 5 minutes. PCR products were analyzed on 2% agarose gel stained with ethidium bromide. The same reaction was used in Real Time PCR reactions.

### Real time quantitative PCR to verify PR1 and PR5 gene expression

The quantitative real-time PCR amplifications were performed using a StepOnePlus Real-Time PCR machine (Applied Biosystems), Rox plus Sybr Green Master Mix 2X (LGC) with the PCR conditions previously described. All experiments were performed in experimental and biological triplicate. The raw data of fluorescence for all runs were imported into the Real-time PCR Miner software in order to determine the Ct value and the PCR efficiency. The most stable reference gene was used for gene expression normalization. The analyses of fold change were performed using qBASE software.

### Sequencing and bioinformatic analysis

Eighteen libraries, consisting of root control and local inoculation, male inflorescence control and systemic inoculation, female inflorescence control and systemic inoculation (3 biological replicates for each), were prepared according to Illumina TruSeq^TM^ RNA Sample Preparation Kit v.3. Four lanes were sequenced in HiSeq 2500 platform with length reads of 100 bp, paired end in Rapid Flow Cell^43^. Six libraries were pooled by lane. Male control and inoculated inflorescences were pooled into one lane, and another lane was used for female inflorescence. Control-inoculated root samples were distributed into two different lanes in order to increase sequencing coverage. High quality reads were obtained after trimming sequences in a sliding window of four nucleotides with phred score over 13 using a Trimmomatic tool^44^. The filtered sequences were mapped to *C. graminicola* reference genome, obtained from Ensembl Fungi database^45^. The genome version used was GCA_000149035.1.23 with the former genus name of *Glomerella*. Bowtie 2^46^ was used to perform the mapping, and unmapped reads were aligned to *Zea mays* genome version AGP v3.23 downloaded from Ensembl Plants database. Gene expression was estimated by counting reads mapped into a feature region using HT-seq^47^. Differentially expressed genes were called up for each experimental condition using the R package DESeq2^48^. Genes with FDR corrected p-value < 0.01 and a minimal fold change of 2 were selected as candidates. Manual annotation of hypothetical genes from transcriptome was performed using InterProScan.

## Electronic supplementary material

Supplementary Information
