# Supplementary material for: Comparative transcriptomic analysis indicates genes associated with local and systemic resistance to Colletotrichum graminicola in maize
Source: Sci Rep. 2017 May 30;7:2483. doi: 10.1038/s41598-017-02298-8 (PMC5449407; doi:10.1038/s41598-017-02298-8)
Supplement: Supplementary file 1 — Supplementary Information [file 41598_2017_2298_MOESM1_ESM.doc]

**Supplementary information**

**Title:** Comparative transcriptomic analysis indicates genes associated with local and systemic resistance to *Colletotrichum graminicola* in maize

**Authors:** Vívian de Jesus Miranda[[1]](#footnote-2), William Farias Porto^1^, Gabriel da Rocha Fernandes^2^, Robert Pogue^1^, Diego Oliveira Nolasco^1,3^, Ana Claudia Guerra Araujo^4^, Luciano Viana Cota^5^, Camila Guimarães de Freitas^1,7^, Simoni Campos Dias^1^, *Octavio Luiz Franco^1,6^

**Supplementary Figure S1**


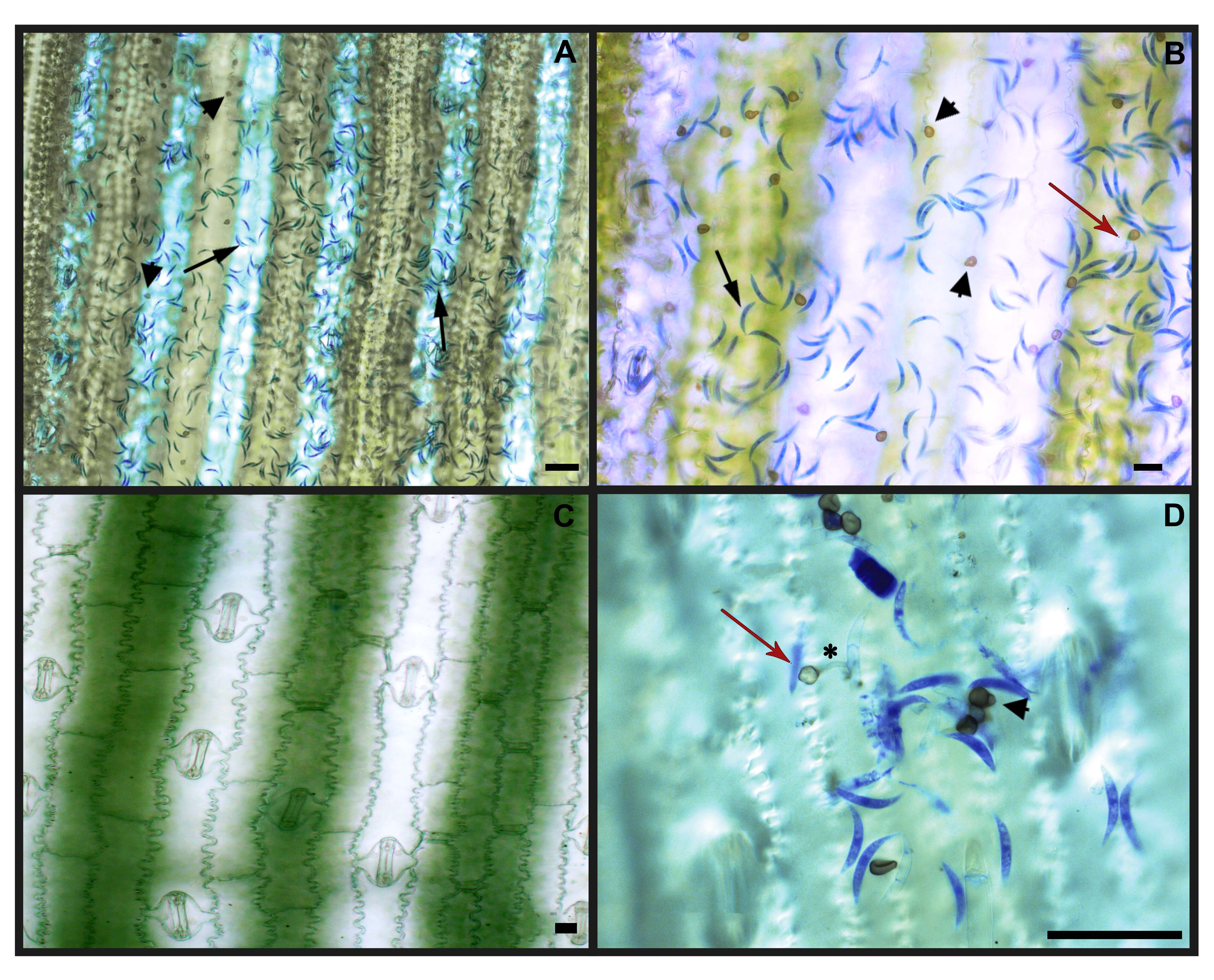


**Differential interference contrast micrograph in maize leaves after 24 h.a.i with *C. graminicola*.** Leaves were cleared and stained with lactophenol cotton blue. In most part of leaves is observed many conidia stained in blue (black arrows) and melanized apressorium (black arrow heads) on the epidermis (A). Small primary hyphae (red arrow) can be observed from the melanotic appressorium (B). Epidermal cells of unifected control leaves without conidia, appressoria and hyphae (C). Image of two daughter cells after mitotic division of a conidia showing germ tube (asterisk), appressorium (arrow head) and small primary hyphae (red arrow) (D). Bars a, c and d = 50 µm and b = 20 µm. All these results were confirmed in accordance with the three biological replicates.

**Supplementary Figure S2**


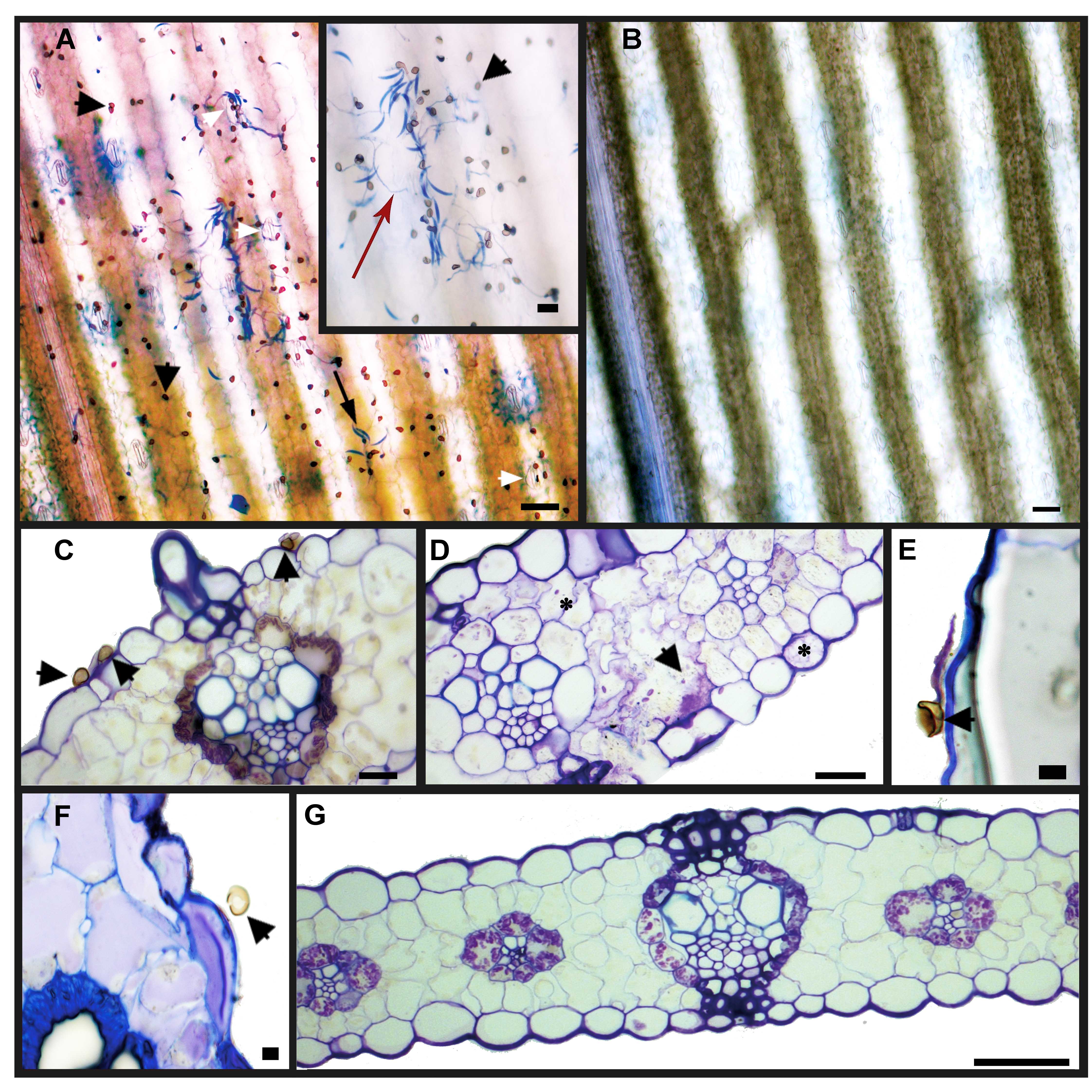


**Micrographs of maize leaves after 36 and 48 h.a.i with *C. graminicola*.** Leaf after bleaching and coloring with cotton blue (A-B) and cross sections after staining with toluidine blue (C-G). Melanized appressoria are observed above the epidermis in a leaf 36 h.a.i with *C. graminicola* (A). A primary hyphae is observed emerging from appressorium and into mesophyll cells. Detail of a leaf region containing superficial appressorium and primary hyphae are highlighted (A). Uninfected control leaf without fungal structures (B). Transversal section of a leaf stained with toluidine blue 36 h.a.i (C). Transversal section of leaf 48 h.a.i showing a hyphae into mesophyll cells (D). Transversal section of a leaf 48 h.a.i showing a longitudinal view of a primary hyphae into the epidermis (E, F). Transversal section of uninfected control leaf 48 h.a.i (G). Black arrow heads: appressoria; white arrow heads: primary hyphae close to stomatal aperture; Red arrows: primary hyphae; * : secondary hyphae. Bars a, c, g = 50 µm; Bar b, d, e = 20 µm; Bar f = 5 µm; Bar h = 10 µm. All these results were confirmed in accordance with the three biological replicates.

**Supplementary Figure S3**


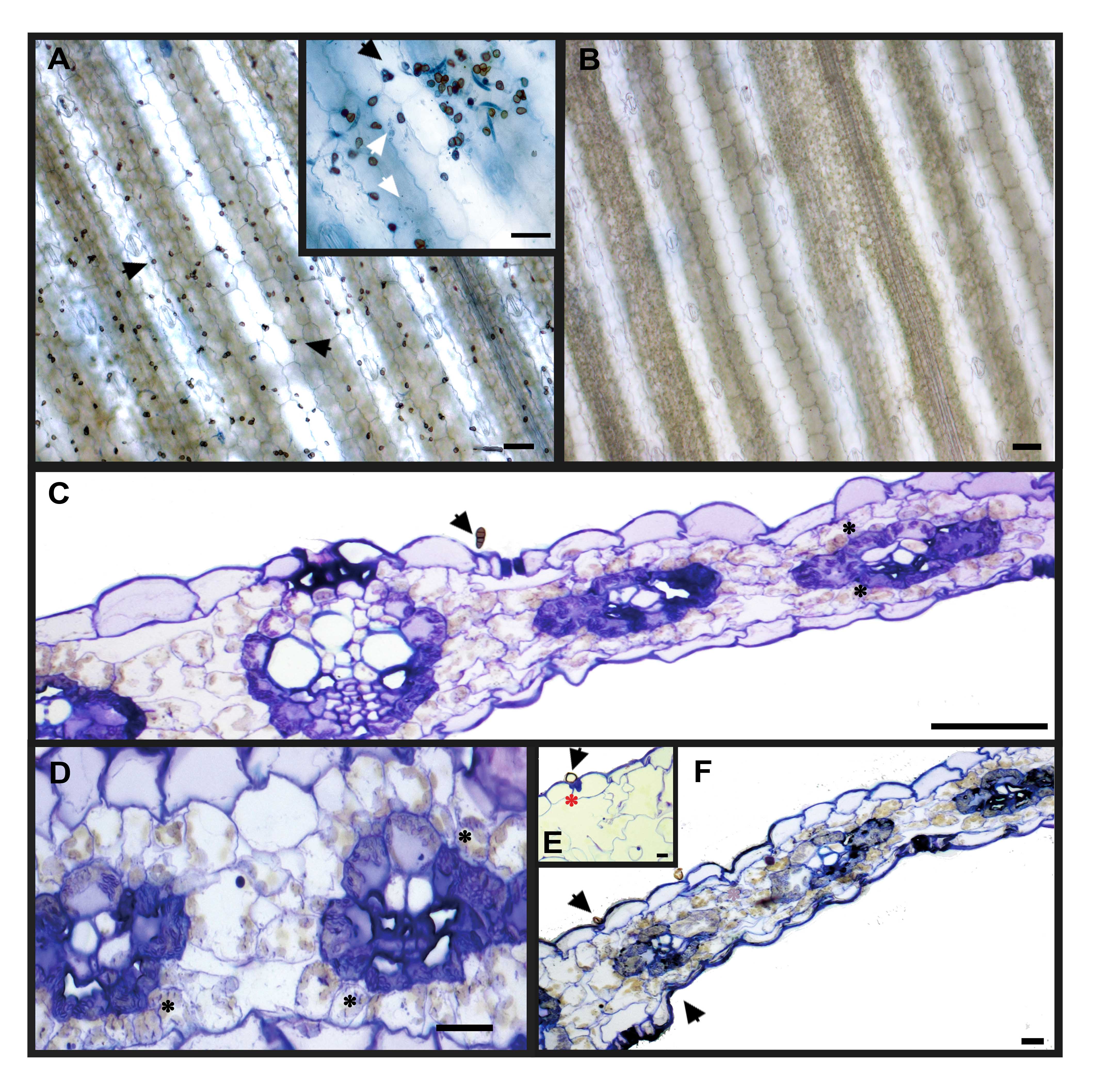


**Light microscopy in brightfield maize leaves infected with *C. graminicola* after 72 h.a.i.** Infected leaf showing several appressoria on the epidermal cells. Detail of an infected leaf region showing appressoria and extensive oxidative stress in the region of fungal infection is highlighted (A). Uninfected control leaf (B). Transversal section of an infected leaf 72 h.a.i showing collapsed mesophyll cells (C). Primary and secondary hyphae in mesophyll cells (D). Lignified papilla formed by an epidermal cell below the appressorium (E). Collapsed mesophyll cells (F). Head of black arrow: appressorium; white arrow heads: signals of oxidative stress, red arrow: primary hyphae, black asterisk: secondary hyphae, red asterisk: lignified papilla. Bars a, b, c, f = 50 μm; bars d, e = 20 μm. All these results were confirmed in accordance with the three biological replicates.

**Supplementary Figure S4**


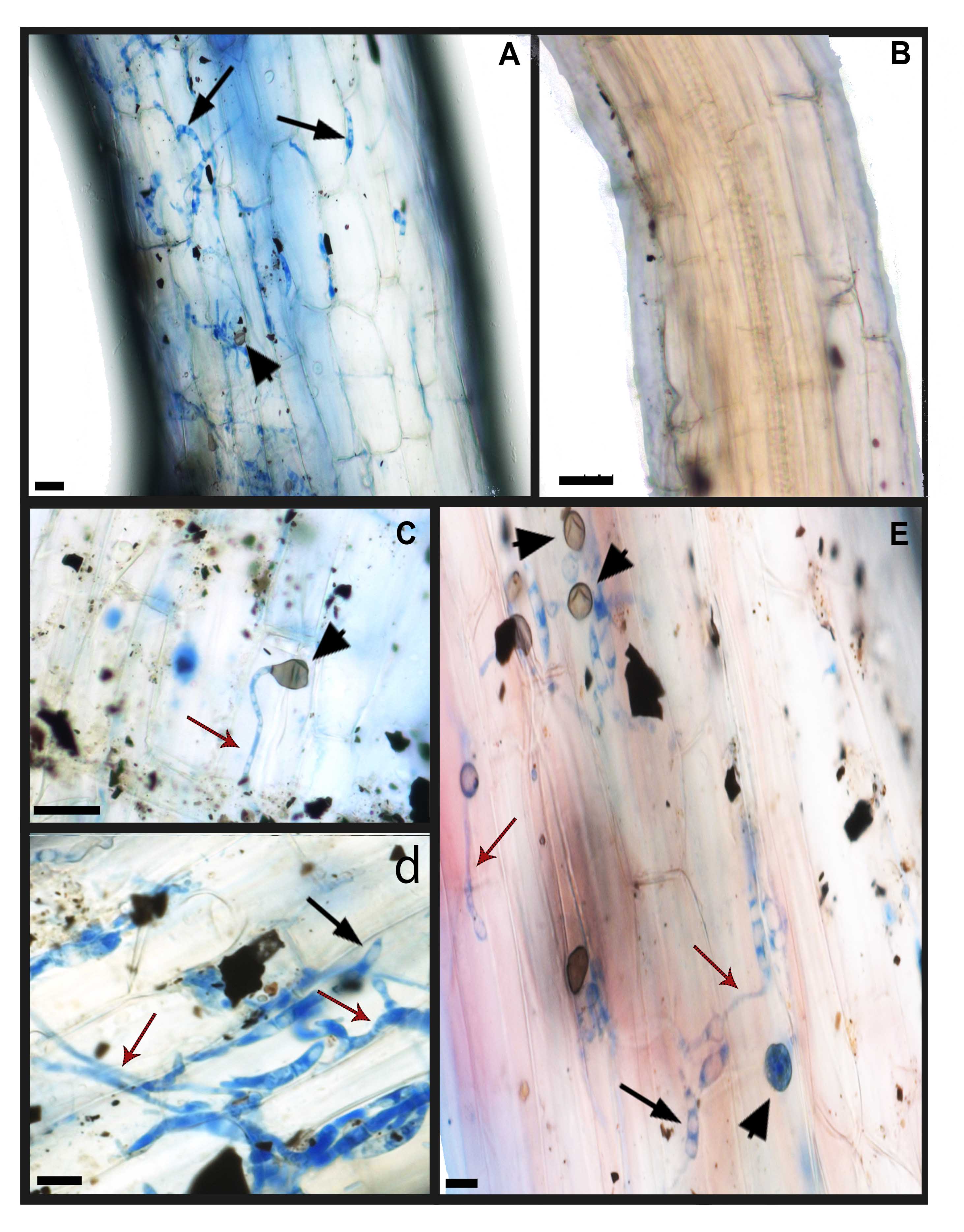


**Light microscopy with DIC in infected maize root with *C. graminicola* after 48 h.a.i.** Conidia adhered to epidermis of root (A). Uninfected control root without fungus (B). Melanotic appressorium with a developing primary hyphae at the root cortex (C). Thicker primary hyphae (D). Melanotic appressorium, appressorium in maturation and primary hyphae are observed in this root section (E). Black arrow: conidia; black arrow head: appressoria, red arrow: primary hyphae. These results were confirmed in accordance with the three biological replicates. Bars a, b, c = 20 μm; Bars d, e = 5 μm.

**Supplementary Fig. S5**


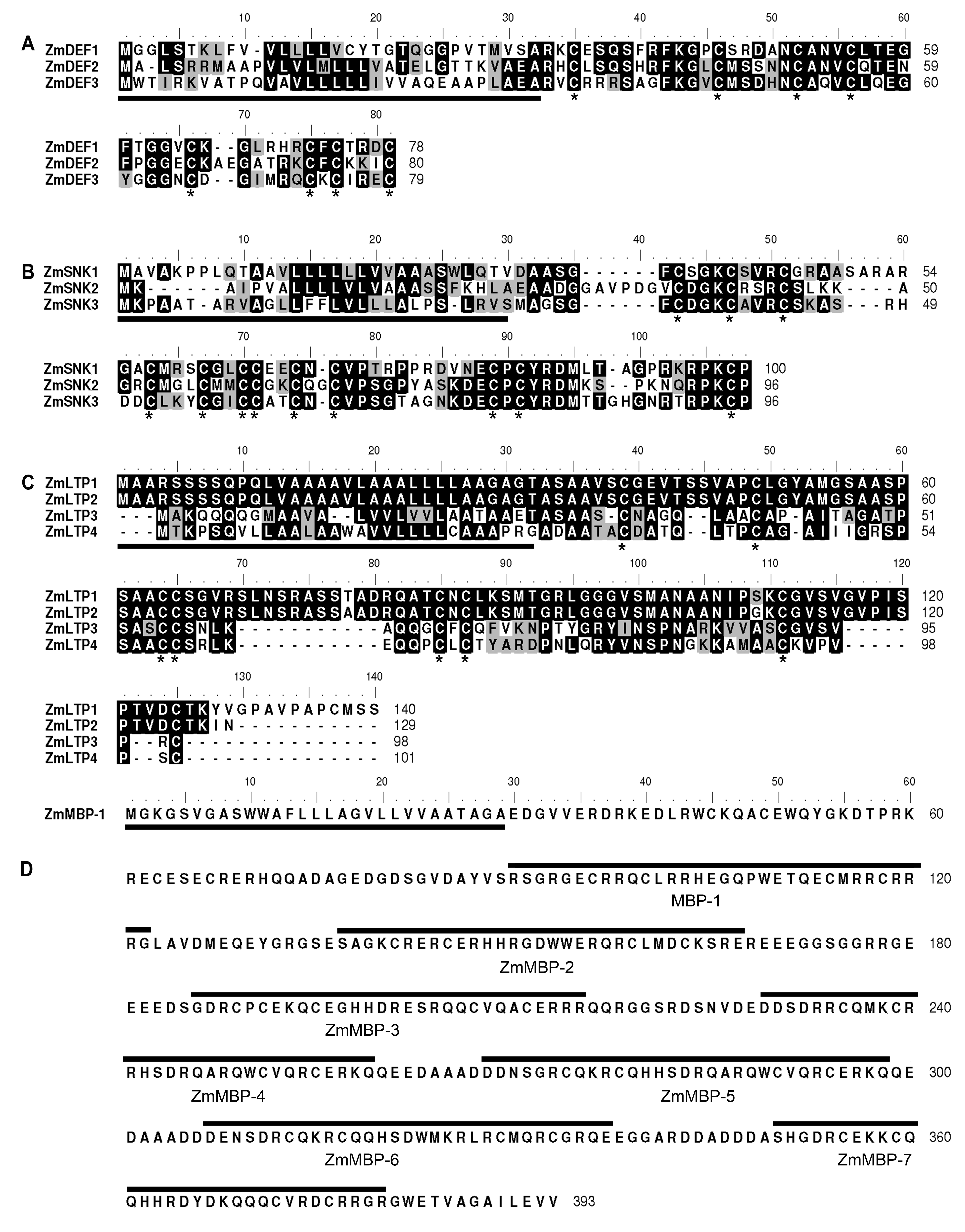


**Multiple sequence alignment of uncharacterized AMPs from NCBI's data base.** Identical residues are showed in black and similar residues in gray. Asterisk represents the conserved cysteines. Black bar below the sequences indicate signal peptide. The bar above the sequences highlight harpinin domains annotated in InterProScan.

**Supplementary Figure S6**


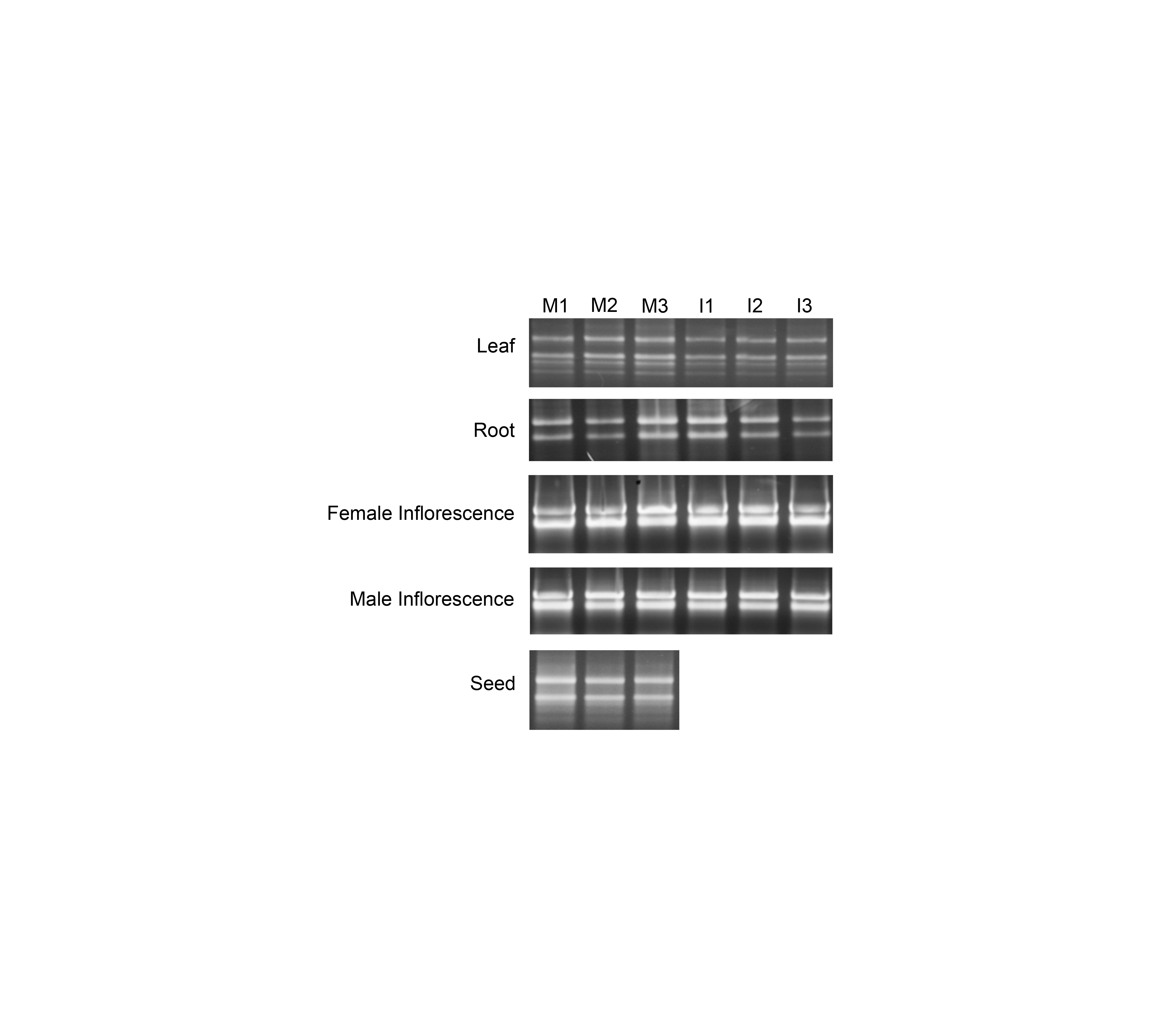


**RNA extraction for different organs and *C.graminicola* inoculation treatments.** Visualization by 1% agarose gel stained with ethidium bromide. All samples showed ratio A260/280 between 1.8 to 2.0 and RIN score above 8.0 measured by Bioanalyzer. M - Mock sample and I - Inoculated sample.

**Supplementary Table S1. Cysteine patterns inside used to screen AMPs from maize database.** Screening was held at NCBI's non-redundant protein database in January 2013. The search was done through PERL scripts, looking for sequences annotated as hypothetical, unnamed, or unknown proteins.

| **Class** | **Cysteine pattern** |
| --- | --- |
| LTP/2S Albumin/ECA1 | C.{6,15}C.{9,31}CC.{8,21}C.C.{13,35}C.{5,18}C |
| LTP/2S Albumin/ECA1 | C.{5,13}C.{14,20}CC.{8,10}C.{10,32}C |
| Defensin | C.{4,25}C.{2,12}C.{3,4}C.{3,17}C.{4,32}C.C.{1,6}C |
| Defensin | C.{2,14}C.{3,5}C.{3,16}C.{4,28}C.C |
| Hevein | C.{1,8}C.{4,5}CC.{5}C.{6}c.{3,5}C.{3,4}C |
| Thionin | C.{3}C.{3,4}C.{4,32}C.{2,3}C.{3,4}C |
| Thionin | CC.{10,11}C.{8,10}C.{5}C.{7,10}C |
| GASA/GAST/Snakin | C.{3}C.{3}C.{7,11}C.{3}C.{2}CC.{2}CC.{2}C.{11}C.{1,2}C |

**Supplementary Table S2. Prediction of antimicrobial activity by CS-AMPPred predictor.**

| **AMP** | **Polynomial** |  | **Radial** |  | **Linear** |  | **Consensus** |
| --- | --- | --- | --- | --- | --- | --- | --- |
| ZmDEF1 | 0,34273656 | AMP | 0,34597987 | AMP | 0,30290465 | AMP | AMP |
| ZmDEF2 | 0,11430099 | AMP | 0,15151923 | AMP | 0,19573442 | AMP | AMP |
| ZmDEF3 | 0,10176331 | AMP | 0,1015175 | AMP | 0,14434111 | AMP | AMP |
| ZmSNK1 | 0,54912984 | AMP | 0,55493189 | AMP | 0,53488148 | AMP | AMP |
| ZmSNK2 | 0,5056798 | AMP | 0,53494869 | AMP | 0,50639833 | AMP | AMP |
| ZmSNK3 | 0,58198072 | AMP | 0,59417674 | AMP | 0,53685459 | AMP | AMP |
| ZmLTP1 | 0,28972596 | AMP | 0,32788614 | AMP | 0,15429697 | AMP | AMP |
| ZmLTP2 | 0,32189075 | AMP | 0,36362893 | AMP | 0,20726748 | AMP | AMP |
| ZmLTP3 | 0,19075168 | AMP | 0,21801207 | AMP | 0,11587402 | AMP | AMP |
| ZmLTP4 | 0,21166462 | AMP | 0,24109293 | AMP | 0,084714277 | AMP | AMP |
| ZmLTP5 | 0,31585121 | AMP | 0,35219694 | AMP | 0,18162908 | AMP | AMP |
| ZmLTP6 | -0,010068952 | AMP | 0,021001585 | AMP | 0,006111784 | Non-AMP | AMP |
| ZmLTP7 | 0,18109063 | AMP | 0,2151877 | AMP | 0,055362433 | Non-AMP | AMP |
| ZmLTP8 | 0,074760835 | AMP | 0,11166982 | AMP | 0,067374471 | Non-AMP | AMP |
| ZmMBP-2 | -0,26125523 | Non-AMP | -0,1570719 | Non-AMP | 0,14788941 | AMP | Non-AMP |
| ZmMBP-3 | -0,59466376 | Non-AMP | -0,39112031 | Non-AMP | -0,058745113 | Non-AMP | Non-AMP |
| ZmMBP-4 | -0,093774379 | Non-AMP | 0,00625191 | AMP | 0,33919198 | AMP | AMP |
| ZmMBP-5 | 0,13944733 | AMP | 0,17528013 | AMP | 0,48434759 | AMP | AMP |
| ZmMBP-6 | -0,69761297 | Non-AMP | -0,46925396 | Non-AMP | -0,13010484 | Non-AMP | Non-AMP |
| ZmMBP-7 | 0,5937008 | AMP | 0,5046278 | AMP | 0,83514548 | AMP | AMP |

**Supplementary Table S3. Mining and quality of RNA-Seq dates.**

| **Sample** | **Reads** | **Yield (Mbases)** | **% of >= Q_30_ Bases (PF)** | **Overall rate of alignment in maize**^a^ **(%) (nº of reads)** | **Overall rate of alignment in fungus**^b^ **(%) (nº of reads)** | **Unmapped in maize or fungus** |
| --- | --- | --- | --- | --- | --- | --- |
| RC 1 | 104,758,980 | 10,476 | 87.4 | 84.08% (88,081,350) | 0.03% (31,428) | 16,646,202 |
| RC 2 | 112,265,426 | 11,227 | 88.1 | 85.95% (96,492,134) | 0.13% (145,945) | 15,627,347 |
| RC 3 | 91,701,690 | 9,170 | 88.5 | 83.72% (76,772,655) | 0.01% (9,170) | 14,919,865 |
| RI 1 | 140,794,260 | 14,080 | 87.6 | 86.30% (121,505,446) | 0.10% (140,794) | 19,148,020 |
| RI 2 | 133,967,746 | 13,397 | 88.6 | 83.94% (112,452,526) | 1.26% (1,687,994) | 19,827,226 |
| RI 3 | 171,808,276 | 17,181 | 88.4 | 86.07% (147,875,383) | 0.55% (944.946) | 22,987,947 |
| FFC1 | 51,863,102 | 5,186 | 90.4 | 80.37% (41,682,375) | 0.03% (15,559) | 10,165,168 |
| FFC2 | 55,634,472 | 5,563 | 90.8 | 84.81% (47,183,596) | 0.01% (5,563) | 8,445,313 |
| FFC3 | 54,744,974 | 5,474 | 90 | 82.08% (44,934,675) | 0.02% (10,949) | 9,799,350 |
| FFI1 | 58,697,366 | 5,870 | 90.2 | 83.00% (48,718,814) | 0.01% (5,870) | 9,972,682 |
| FFI2 | 56,814,718 | 5,681 | 90.4 | 83.20% (47,269,845) | 0.02% (11,363) | 9,533,510 |
| FFI3 | 55,736,268 | 5,574 | 90.4 | 84.22% (46,941,085) | 0.01% (5,574) | 8,789,609 |
| FMC1 | 46,588,230 | 4,659 | 92.3 | 80.62% (37,559,431) | 0.00% | 9,028,799 |
| FMC2 | 43,916,754 | 4,392 | 90.3 | 80.92% (35,537,437) | 0.01% (4,392) | 8,374,925 |
| FMC3 | 48,078,342 | 4,808 | 91.2 | 81.97% (39,409,817) | 0.01% (4,808) | 8,663,717 |
| FMI1 | 45,873,864 | 4,587 | 92.4 | 80.72% (37,029,383) | 0.00% | 8,844,481 |
| FMI2 | 46,790,220 | 4,679 | 92.1 | 81.39% (38,082,560) | 0.01% (4,679) | 8,702,981 |
| FMI3 | 43,990,072 | 4,399 | 90 | 85.36% (37,549,925) | 0.07% (30,793) | 6,409,354 |

**Supplementary Table S4. Primers used in this study.**

| **Gene Name** | **Symbol** | **Sequences**  **(Foward / Reverse)** | **Amplicon**  **size** | **Location^*^** | |
| --- | --- | --- | --- | --- | --- |
| *leunig* | *LUG* | TCCAGTGCTACAGGGAAGGT | 152 | - | Housekeeping gene |
|  |  | GCGTCATGTGGTCATTTTTG |  |  |
| *glyceraldehyde 3-phosphate dehydrogenase* | *GAPC* | GCATCAGGAACCCTGAGGAA  CATGGGTGCATCTTTGCTTG | 158 | - |
| *membrane protein* | *MEP* | CCATCTGTCTGGGTCAGGAT | 129 | 3'UTR |
|  |  | TTTGATGCTCCAGGCTTACC |  |  |
| *actin 1* | *ACT1* | GATTCCTGGGATTGCCGAT | 152 | - |
|  |  | TCTGCTGCTGAAAAGTGCTGAG |  |  |
| *pathogenesis-related protein 1* | *PR1* | CCTACGGCGAGAACCTCTT | 94 | - | Stress marker gene |
|  |  | TCGTAGTACTGCTTCTCGGACA |  |  |
| *ribonuclease* | *PR10.1* | CAGCTGGACTGTTGAGATCG  GTGTGCCAGTCCATCACG | 75 | - |
| *thaumatin* | *PR5* | CTGGCCGAGTTCACCATC | 77 | - |
|  |  | GCCATGGCGAGGTTGTAG |  |  |
| *defensin 1* | *ZmDEF1* | TCTCTCCACCAAGCTTTTCG | 129 | DE | Potential novels AMPs from NCBI's databse |
|  |  | GAGCAAGGTCCCTTGAAGC |  |  |
| *defensin 2* | *ZmDEF2* | GTCCTCGTCCTCATGCTCCT | 121 | DE |
|  |  | AGTTGTTGCTGCTCATGCAC |  |  |
| *defensin 3* | *ZmDEF3* | ATGTGGACGATCAGGAAGGT | 143 | DE |
|  |  | GACATGCAGACCCCCTTG |  |  |
| *snakin 1* | *ZmSKN1* | CTGCGAGGAGTGCAACTG | 106 | ME |
|  |  | GCACTTGGGCCTCTTCCT |  |  |
| *snakin 2* | *ZmSKN2* | CTCTCCTGCTCCTCGTCCT | 130 | DE |
|  |  | GCCTTCTTCAGCGAGCAC |  |  |
| *snakin 3* | *ZmSKN3* | TTCTTCCTCGTCCTCCTCCT | 125 | DE |
|  |  | TACTTGAGGCAGTCGTCGTG |  |  |
| *lipid transfer protein 1* | *ZmLTP1* | GCTGGCTTCGTCCGCTTC | 164 | ME |
|  |  | GCTGTTGAGGCTCTTGACG |  |  |
| *lipid transfer protein 2 lipídeo* | *ZmLTP2* | TCGTGAAGAACCCCACCTAC | 121 | ME |
|  |  | TACATGCATGCACAGCACAC |  |  |
| *lipid transfer protein 3 lipídeo* | *ZmLTP3* | GCTCAAGCTTGCTCCTTGC | 129 | ME |
|  |  | GTGTCGGACAGCATGACG |  |  |
| *lipid transfer protein 4 lipídeo* | *ZmLTP4* | AACTGCCTCAAGAGCATGAC | 134 | ME |
|  |  | CCTACGTACTTGGTGCAGTCG |  |  |
| *lipid transfer protein 5 lipídeo* | *ZmLTP5* | GTGTGCGCTTCTGTCTGTGT | 129 | ME |
|  |  | CTTCCACGACTCACCACTCA |  |  |
| *lipid transfer protein 6 lipídeo* | *ZmLTP6* | CCATCATCATCGGGAGGTC | 133 | ME |
|  |  | ATGGCCTTCTTGCCGTTG |  |  |
| *lipid transfer protein 7 lipídeo* | *ZmLTP7* | CGTCTAGTGTCCCTCCGTTC | 128 | ME |
|  |  | AGAGAGCCAGCAAAGCAAAC |  |  |
| *lipid transfer protein 8 de lipídeo* | *ZmLTP8* | CCAAGGATTTGGCTGGTTAG | 125 | ME |
|  |  | AACGCAATCCAACACAAACA |  |  |

* DE - different exon; SE - same exon

**Supplementary Table S5. Differentially expressed genes in *C. graminicola* inoculated-root relative to mock-plant** using RNA-Seq data

| **ID (maizeGDB)** | **Description** | **Base Mean** | **Log_2_ Fold Change** | ***P-value*** | ***P-adj*** |
| --- | --- | --- | --- | --- | --- |
| GRMZM2G005155 | MADS transcription fator | 96,3523 | -2,702 | 3,11E-09 | 2,50E-06 |
| GRMZM2G007256 | Adhesive/proline-rich protein | 1750,9843 | -9,054 | 2,79E-125 | 8,53E-121 |
| GRMZM2G048801 | Putative leucine-rich repeat receptor-like protein kinase family protein | 347,5615 | 1,06 | 3,22E-07 | 0,000147 |
| GRMZM2G049781 | Putative heavy metal transport//detoxification superfamily protein | 101,3914 | 2,367 | 1,90E-09 | 1,62E-06 |
| GRMZM2G079638 | Anther-specific protein SF18 | 1323,7285 | -1,206 | 1,85E-05 | 0,005764 |
| GRMZM2G096358 | MYB transcription factor | 110,4966 | 2,281 | 7,42E-08 | 3,91E-05 |
| GRMZM2G125704 | G2-like transcription factor | 77,8756 | -2,172 | 5,84E-08 | 3,19E-05 |
| GRMZM2G112968 | Putative nuclease | 1325,9186 | 1,434 | 6,11E-09 | 4,34E-06 |
| GRMZM2G134502 | Fiber annexin | 407,0391 | -1,083 | 3,64E-06 | 0,001373 |
| GRMZM2G140754 | Alanyl-tRNA synthetase | 722,5981 | 2,276 | 9,23E-11 | 9,10E-08 |
| GRMZM2G149923 | Calmodulin | 519,3022 | 1,637 | 8,94E-09 | 6,07E-06 |
| GRMZM2G151738 | Leucine-rich repeat (LRR) family protein | 427,2012 | 1,363 | 2,95E-05 | 0,008765 |
| GRMZM2G161891 | Glutathione S-transferase GST 35 | 342,5341 | 1,725 | 1,12E-08 | 7,13E-06 |
| GRMZM2G168404 | Putative cytochrome P450 superfamily protein | 69,0169 | -2,75 | 1,77E-08 | 1,08E-05 |
| GRMZM2G168552 | Bundle sheath cell specific protein 1 | 8490,6421 | -1,29 | 2,27E-08 | 1,31E-05 |
| GRMZM2G168956 | 3-ketoacyl-CoA synthase | 157,3888 | 1,781 | 1,64E-06 | 0,000659 |
| GRMZM2G487328 | Serine/threonine-protein kinase NAK | 98,2857 | -1,302 | 1,03E-05 | 0,003446 |
| AC194402.2_FG003 | - | 80,0033 | 3,861 | 7,04E-19 | 1,66E-15 |
| AC196954.2_FG012 |  | 14,5029 | -2,68 | 4,17E-07 | 0,000187 |
| AC199843.4_FG007 | Uncharacterized protein | 10,5169 | 2,234 | 3,16E-05 | 0,00904 |
| AC212809.2_FG002 | - | 38,7844 | 3,365 | 2,15E-11 | 2,19E-08 |
| AC218998.2_FG007 | Uncharacterized protein | 1419,1662 | 6,684 | 8,27E-53 | 6,32E-49 |
| AC218998.2_FG011 | Uncharacterized protein | 2584,3513 | 5,995 | 7,17E-73 | 1,10E-68 |
| GRMZM2G029211 | Uncharacterized protein | 33,5388 | -1,764 | 1,25E-05 | 0,004096 |
| GRMZM2G005536 | Uncharacterized protein | 359,0741 | 1,052 | 1,75E-05 | 0,005569 |
| GRMZM2G032190 | Uncharacterized protein | 3683,8107 | 1,101 | 4,98E-06 | 0,00179 |
| GRMZM2G032551 | Uncharacterized protein | 23,5198 | 3,123 | 3,99E-09 | 3,12E-06 |
| GRMZM2G034611 | Uncharacterized protein | 679,804 | 2,375 | 3,89E-24 | 1,98E-20 |
| GRMZM2G037617 | - | 523,7917 | 2,556 | 9,41E-17 | 1,65E-13 |
| GRMZM2G041171 | Uncharacterized protein | 13,1341 | 2,629 | 9,23E-07 | 0,000386 |
| GRMZM2G051522 | Uncharacterized protein | 737,6519 | 2,483 | 1,53E-08 | 9,53E-06 |
| GRMZM2G054415 | Uncharacterized protein | 51,9114 | 3,724 | 1,14E-12 | 1,34E-09 |
| GRMZM2G058884 | - | 411,3892 | 2,356 | 2,85E-08 | 1,58E-05 |
| GRMZM2G061795 | Uncharacterized protein | 337,2671 | 1,709 | 4,80E-06 | 0,001744 |
| GRMZM2G063798 | - | 2133,4509 | 1,405 | 5,22E-09 | 3,80E-06 |
| GRMZM2G064139 | - | 73,6081 | 2,112 | 3,77E-06 | 0,001403 |
| GRMZM2G070041 | Uncharacterized protein | 24,4931 | 3,112 | 4,73E-09 | 3,61E-06 |
| GRMZM2G077546 | Uncharacterized protein | 238,9472 | 2,34 | 4,25E-13 | 5,19E-10 |
| GRMZM2G079219 | Uncharacterized protein | 390,917 | 3,038 | 5,36E-23 | 2,05E-19 |
| GRMZM2G088293 | - | 18,9545 | 2,537 | 2,32E-06 | 0,000897 |
| GRMZM2G092474 | Putative uncharacterized protein | 850,258 | 1,131 | 3,04E-05 | 0,00885 |
| GRMZM2G093072 | Uncharacterized protein | 228,7128 | 2,923 | 4,75E-14 | 6,90E-11 |
| GRMZM2G094808 | Uncharacterized protein | 22,2002 | 2,215 | 2,87E-05 | 0,008696 |
| GRMZM2G102015 | Uncharacterized protein | 116,0267 | 4,66 | 1,70E-28 | 1,04E-24 |
| GRMZM2G103914 | Uncharacterized protein | 20,9631 | 2,774 | 2,24E-07 | 0,000107 |
| GRMZM2G107645 | Uncharacterized protein | 361,1927 | 1,308 | 8,24E-08 | 4,20E-05 |
| GRMZM2G107688 | - | 48,3181 | 4,703 | 1,39E-22 | 4,72E-19 |
| GRMZM2G113421 | Uncharacterized protein | 277,4918 | 2,295 | 5,42E-14 | 7,53E-11 |
| GRMZM2G113652 | Uncharacterized protein | 374,7543 | 1,51 | 2,65E-07 | 0,000125 |
| GRMZM2G117942 | Uncharacterized protein; Win2 | 1222,1042 | 2,511 | 1,98E-07 | 9,59E-05 |
| GRMZM2G127499 | Putative uncharacterized protein | 117,382 | 1,963 | 2,08E-06 | 0,000814 |
| GRMZM2G129196 | Uncharacterized protein | 24,3598 | 3,521 | 1,22E-11 | 1,33E-08 |
| GRMZM2G134340 | Uncharacterized protein | 2141,7648 | 1,556 | 1,38E-10 | 1,32E-07 |
| GRMZM2G147210 | - | 433,5203 | 1,876 | 1,61E-05 | 0,005168 |
| GRMZM2G148626 | Uncharacterized protein | 274,6908 | 1,32 | 2,01E-06 | 0,000799 |
| GRMZM2G168985 | - | 33,0451 | -2,421 | 6,58E-06 | 0,00231 |
| GRMZM2G169013 | Uncharacterized protein | 292,6528 | -2,186 | 1,52E-10 | 1,41E-07 |
| GRMZM2G172386 | Uncharacterized protein | 47,924 | 2,677 | 5,10E-09 | 3,80E-06 |
| GRMZM2G177218 | Hypro1; Putative uncharacterized protein hypro1 | 309,2967 | -1,221 | 3,50E-05 | 0,009623 |
| GRMZM2G181266 | Uncharacterized protein | 9,9674 | 2,241 | 2,98E-05 | 0,008765 |
| GRMZM2G301389 | - | 84,4878 | -4,235 | 1,26E-17 | 2,56E-14 |
| GRMZM2G333045 | Uncharacterized protein | 178,6281 | 1,941 | 2,12E-12 | 2,40E-09 |
| GRMZM2G337109 | - | 173,8194 | 2,92 | 5,10E-20 | 1,30E-16 |
| GRMZM2G359822 | - | 776,7902 | 1,872 | 3,17E-05 | 0,00904 |
| GRMZM2G363554 | - | 192,2402 | -1,015 | 8,74E-07 | 0,000376 |
| GRMZM2G370999 | Uncharacterized protein | 192,5408 | 2,049 | 3,61E-05 | 0,009849 |
| GRMZM2G371651 | Uncharacterized protein | 35,9295 | 3,006 | 7,40E-09 | 5,14E-06 |
| GRMZM2G376661 | - | 594,3281 | 1,486 | 2,77E-07 | 0,000128 |
| GRMZM2G392026 | - | 15,6044 | 3,025 | 1,08E-08 | 7,05E-06 |
| GRMZM2G392076 | - | 991,7664 | 2,094 | 2,11E-09 | 1,74E-06 |
| GRMZM2G403652 | Uncharacterized protein | 106,3147 | 1,733 | 8,59E-07 | 0,000375 |
| GRMZM2G411569 | Uncharacterized protein | 591,1662 | -2,812 | 9,69E-17 | 1,65E-13 |
| GRMZM2G445854 | Uncharacterized protein | 603,5749 | 6,807 | 5,65E-64 | 5,75E-60 |
| GRMZM2G448927 | - | 878,8629 | 1,619 | 4,19E-06 | 0,001543 |
| GRMZM2G454511 | - | 23,7653 | -2,61 | 1,18E-06 | 0,000487 |
| GRMZM2G472827 | - | 99,7531 | 2,696 | 1,62E-11 | 1,71E-08 |
| GRMZM2G480621 | - | 27,972 | 3,762 | 2,17E-13 | 2,76E-10 |
| GRMZM2G509699 | - | 20,3698 | -2,349 | 1,38E-06 | 0,000561 |
| GRMZM2G527017 | Uncharacterized protein | 26,4034 | 2,863 | 7,71E-08 | 3,99E-05 |
| GRMZM2G579719 | Uncharacterized protein | 277,6375 | -1,433 | 1,41E-07 | 6,96E-05 |
| GRMZM5G804477 | - | 83,0614 | -4,83 | 4,90E-22 | 1,50E-18 |
| GRMZM5G809663 | - | 26,8667 | 3,853 | 3,19E-14 | 5,12E-11 |
| GRMZM5G813244 | - | 34,4725 | 1,914 | 8,05E-06 | 0,002734 |
| GRMZM5G823824 | Uncharacterized protein | 47,9661 | -4,658 | 2,50E-23 | 1,09E-19 |
| GRMZM5G834759 | - | 10,7137 | -2,337 | 1,34E-05 | 0,004345 |
| GRMZM5G839762 | - | 46,9298 | 3,872 | 7,85E-14 | 1,04E-10 |
| GRMZM5G880063 | - | 15,6044 | 3,025 | 1,08E-08 | 7,05E-06 |
| GRMZM5G885872 | Uncharacterized protein | 39,0068 | 2,428 | 5,46E-06 | 0,001941 |
| GRMZM5G885938 | Putative uncharacterized protein | 64,8692 | -4,452 | 2,35E-18 | 5,12E-15 |
| GRMZM5G887396 | Uncharacterized protein | 14,1733 | -3,198 | 1,37E-09 | 1,19E-06 |
| GRMZM5G899582 | Putative uncharacterized protein | 66,523 | -4,369 | 1,54E-17 | 2,93E-14 |
| GRMZM2G064344 | Uncharacterized protein | 7,8537 | -2,239 | 2,98E-05 | 0,008765 |
| GRMZM2G073324 | Uncharacterized protein | 1204,2953 | -1,25 | 2,52E-05 | 0,007711 |
| GRMZM2G080156 | Uncharacterized protein | 89,3622 | -4,728 | 7,53E-22 | 2,09E-18 |
| GRMZM2G086430 | Uncharacterized protein | 476,6208 | -1,136 | 9,19E-07 | 0,000386 |
| GRMZM2G108875 | - | 10,769 | -2,703 | 4,52E-07 | 0,0002 |
| GRMZM2G109812 | - | 66,0458 | -2,213 | 1,01E-07 | 5,05E-05 |
| GRMZM2G112942 | - | 812,6731 | -1,203 | 1,12E-09 | 1,01E-06 |
| GRMZM2G125679 | - | 103,6819 | -3,348 | 4,47E-14 | 6,82E-11 |
| GRMZM2G143258 | Uncharacterized protein | 2182,2846 | -1,112 | 2,22E-08 | 1,31E-05 |
| GRMZM2G167658 | - | 42,4719 | -2,801 | 6,16E-08 | 3,30E-05 |

**Supplementary Table S6. Functional re-annotation of differentially expressed genes between mock-plants and *C. graminicola* inoculated-root**

| **ID (maizeGDB)** | **InterProScan annotation** | **Gene ontology**  **(biological process)** | **Gene ontology**  **(molecular function)** | **Gene ontology**  **(cellular component)** |
| --- | --- | --- | --- | --- |
| GRMZM2G005155 | IPR002100 [DOMAIN] - Transcription factor, MADS-box | transcription, DNA-templated | DNA binding; transcription factor activity, sequence-specific DNA binding | nucleus |
| GRMZM2G007256 | IPR028144 [DOMAIN] - Cysteine-rich transmembrane CYSTM domain | - | - | - |
| GRMZM2G048801 | IPR013210 [DOMAIN] - Leucine-rich repeat-containing N-terminal, plant-type | - | ATP binding; protein serine/threonine kinase activity | integral component of membrane |
| GRMZM2G049781 | IPR006121 [DOMAIN] - Heavy metal-associated domain, HMA | metal ion transport | metal ion binding | - |
| GRMZM2G079638 | - | - | - | - |
| GRMZM2G096358 | IPR017930 [DOMAIN] - Myb domain | - | DNA binding | - |
| GRMZM2G125704 | - | regulation of transcription, DNA-templated; transcription, DNA-templated | DNA binding | nucleus |
| GRMZM2G112968 | IPR003154 [FAMILY] - S1/P1 nuclease | DNA catabolic process | endonuclease activity; nucleic acid binding | - |
| GRMZM2G134502 | IPR018502 [REPEAT] - Annexin repeat | - | calcium-dependent phospholipid binding; calcium ion binding | - |
| GRMZM2G140754 | IPR018165 [DOMAIN] - Alanyl-tRNA synthetase, class IIc, core domain | - | nucleotide binding | - |
| GRMZM2G149923 | IPR011992 [DOMAIN] - EF-hand domain pair | - | calcium ion binding | - |
| GRMZM2G151738 | IPR032675 [DOMAIN] - Leucine-rich repeat domain, L domain-like | - | - | - |
| GRMZM2G161891 | IPR004045 [DOMAIN] - Glutathione S-transferase, N-terminal | - | transferase activity | - |
| GRMZM2G168404 | IPR001128 [FAMILY] - Cytochrome P450 |  | heme binding; iron ion binding;oxidoreductase activity, acting on paired donors, with incorporation or reduction of molecular oxygen |  |
| GRMZM2G168552 | IPR003496 [FAMILY] - ABA/WDS induced protein | response to stress | - | - |
| GRMZM2G168956 | IPR016039 [DOMAIN] - Thiolase-like | fatty acid biosynthetic process | transferase activity, transferring acyl groups other than amino-acyl groups | integral component of membrane |
| GRMZM2G487328 | IPR013320 [DOMAIN] - Concanavalin A-like lectin/glucanase domain / IPR000719 [DOMAIN] - Protein kinase domain | - | ATP binding; protein serine/threonine kinase activity | integral component of membrane |
| AC194402.2_FG003 | - | - | - | - |
| AC196954.2_FG012 | IPR004864 [DOMAIN] - Late embryogenesis abundant protein, LEA-14 | - | - | - |
| AC199843.4_FG007 | IPR001878 [DOMAIN] - Zinc finger, CCHC-type | - | nucleic acid binding; zinc ion binding | - |
| AC212809.2_FG002 | - | - | - | - |
| AC218998.2_FG007 | IPR011009 [DOMAIN] - Protein kinase-like domain / IPR001229 [DOMAIN] - Jacalin-like lectin domain | - | ATP binding; protein kinase activity | - |
| AC218998.2_FG011 | IPR008930 [DOMAIN] - Terpenoid cyclases/protein prenyltransferase alpha-alpha toroid | - | magnesium ion binding; terpene synthase activity | - |
| GRMZM2G029211 | IPR032675 [DOMAIN] - Leucine-rich repeat domain, L domain-like | - | - | - |
| GRMZM2G005536 | IPR019038 [FAMILY] - DNA polymerase subunit Cdc27 | - | - | - |
| GRMZM2G032190 | - | - | - | - |
| GRMZM2G032551 | IPR007658 [FAMILY] - Protein of unknown function DUF594 | - | - | integral component of membrane |
| GRMZM2G034611 | IPR013320 [DOMAIN] - Concanavalin A-like lectin/glucanase domain / IPR002290 [DOMAIN] - Serine/threonine/dual specificity protein kinase, catalytic domain | - | ATP binding; protein serine/threonine kinase activity | integral component of membrane |
| GRMZM2G037617 | IPR030614 [FAMILY] - Serine/threonine-protein kinase Aurora-3 | chromosome segregation; microtubule cytoskeleton organization; mitotic cell cycle; regulation of cytokinesis | ATP binding; protein kinase activity | - |
| GRMZM2G041171 | IPR004252 [FAMILY] - Probable transposase, Ptta/En/Spm, plant | - | - | - |
| GRMZM2G051522 | IPR025422 [DOMAIN] - Transcription factor TGA like domain | transcription, DNA-templated | sequence-specific DNA binding | - |
| GRMZM2G054415 | - | - | - | integral component of membrane |
| GRMZM2G058884 | - | - | - | - |
| GRMZM2G061795 | IPR029044 [DOMAIN] - Nucleotide-diphospho-sugar transferases | biosynthetic process | nucleotidyltransferase activity | - |
| GRMZM2G063798 | IPR000462 [FAMILY] - CDP-alcohol phosphatidyltransferase | phospholipid biosynthetic process | phosphotransferase activity, for other substituted phosphate groups | integral component of membrane |
| GRMZM2G064139 | - | - | - | integral component of membrane |
| GRMZM2G070041 | IPR004252 [FAMILY] - Probable transposase, Ptta/En/Spm, plant | - | - | - |
| GRMZM2G077546 | IPR003663 [FAMILY] - Sugar/inositol transporter | - | substrate-specific transmembrane transporter activity | integral component of membrane |
| GRMZM2G079219 | IPR013320 [DOMAIN] - Concanavalin A-like lectin/glucanase domain / IPR002290 [DOMAIN] - Serine/threonine/dual specificity protein kinase, catalytic domain | - | ATP binding; protein serine/threonine kinase activity | integral component of membrane |
| GRMZM2G088293 | IPR004252 [FAMILY] - Probable transposase, Ptta/En/Spm, plant | - | - | - |
| GRMZM2G092474 | IPR001938 [FAMILY] - Thaumatin | - | - | - |
| GRMZM2G093072 | IPR025287 [DOMAIN] - Wall-associated receptor kinase, galacturonan-binding domain / IPR002290 [DOMAIN] - Serine/threonine/dual specificity protein kinase, catalytic domain | - | ATP binding; polysaccharide binding; protein serine/threonine kinase activity | - |
| GRMZM2G094808 | - | - | - | - |
| GRMZM2G102015 | IPR001977 [FAMILY] - Dephospho-CoA kinase | coenzyme A biosynthetic process | ATP binding; dephospho-CoA kinase activity | - |
| GRMZM2G103914 | - | - | - | - |
| GRMZM2G107645 | IPR030614 [FAMILY] - Serine/threonine-protein kinase Aurora-3 | chromosome segregation; microtubule cytoskeleton organization; mitotic cell cycle; regulation of cytokinesis | ATP binding; protein kinase activity | - |
| GRMZM2G107688 | IPR000223 [FAMILY] - Peptidase S26A, signal peptidase I | - | serine-type peptidase activity | integral component of membrane |
| GRMZM2G113421 | IPR013320 [DOMAIN] - Concanavalin A-like lectin/glucanase domain / IPR002290 [DOMAIN] - Serine/threonine/dual specificity protein kinase, catalytic domain | - | ATP binding; polysaccharide binding; protein serine/threonine kinase activity | - |
| GRMZM2G113652 | IPR027640 [FAMILY] - Kinesin-like protein | microtubule-based movement | ATP binding; microtubule motor activity | kinesin complex; microtubule; integral component of membrane |
| GRMZM2G117942 | IPR001153 [DOMAIN] - Barwin domain | defense response to bacterium; defense response to fungus | - | - |
| GRMZM2G127499 | - | - | - | - |
| GRMZM2G129196 | IPR004252 [FAMILY] - Probable transposase, Ptta/En/Spm, plant | - | - | - |
| GRMZM2G134340 | - | - | - | - |
| GRMZM2G147210 | IPR012336 [DOMAIN] - Thioredoxin-like fold | cell redox homeostasis | - | cell |
| GRMZM2G148626 | IPR019038 [FAMILY] - DNA polymerase subunit Cdc27 | DNA replication | - | nucleus |
| GRMZM2G168985 | IPR001480 [DOMAIN] - Bulb-type lectin domain / IPR002290 [DOMAIN] - Serine/threonine/dual specificity protein kinase, catalytic domain | recognition of pollen | ATP binding; protein kinase activity | - |
| GRMZM2G169013 | - | - | - | - |
| GRMZM2G172386 | IPR032872 [DOMAIN] - Wall-associated receptor kinase, C-terminal / IPR013320 [DOMAIN] - Concanavalin A-like lectin/glucanase domain | - | ATP binding; protein serine/threonine kinase activity | integral component of membrane |
| GRMZM2G177218 | IPR002659 [FAMILY] - Glycosyl transferase, family 31 | protein glycosylation | galactosyltransferase activity | Golgi apparatus; integral component of membrane |
| GRMZM2G181266 | IPR007482 [FAMILY] - Protein-tyrosine phosphatase-like, PTPLA | - | - | integral component of membrane |
| GRMZM2G301389 | IPR007482 [FAMILY] - Protein-tyrosine phosphatase-like, PTPLA / IPR007482 [FAMILY] - Protein-tyrosine phosphatase-like, PTPLA | - | - | - |
| GRMZM2G333045 | IPR025287 [DOMAIN] - Wall-associated receptor kinase, galacturonan-binding domain / IPR002290 [DOMAIN] - Serine/threonine/dual specificity protein kinase, catalytic domain | - | ATP binding; polysaccharide binding; protein serine/threonine kinase activity | - |
| GRMZM2G337109 | IPR030614 [FAMILY] - Serine/threonine-protein kinase Aurora-3 | chromosome segregation; microtubule cytoskeleton organization; mitotic cell cycle; regulation of cytokinesis | ATP binding; protein kinase activity | - |
| GRMZM2G359822 | IPR002480 [FAMILY] - DAHP synthetase, class II | aromatic amino acid family biosynthetic process | 3-deoxy-7-phosphoheptulonate synthase activity | - |
| GRMZM2G363554 | IPR002213 [FAMILY] - UDP-glucuronosyl/UDP-glucosyltransferase | - | transferase activity, transferring hexosyl groups | - |
| GRMZM2G370999 | - | - | - | - |
| GRMZM2G371651 | - | - | - | - |
| GRMZM2G376661 | IPR002403 [FAMILY] - Cytochrome P450, E-class, group IV |  | heme binding; iron ion binding; monooxygenase activity; oxidoreductase activity, acting on paired donors, with incorporation or reduction of molecular oxygen |  |
| GRMZM2G392026 | - | - | - | - |
| GRMZM2G392076 | IPR002885 [REPEAT] - Pentatricopeptide repeat | - | - | - |
| GRMZM2G403652 | IPR012336 [DOMAIN] - Thioredoxin-like fold / IPR010987 [DOMAIN] - Glutathione S-transferase, C-terminal-like | - | - | - |
| GRMZM2G411569 | IPR004856 [FAMILY] - Glycosyl transferase, ALG6/ALG8 | - | transferase activity, transferring hexosyl groups | endoplasmic reticulum membrane |
| GRMZM2G445854 | IPR008930 [DOMAIN] - Terpenoid cyclases/protein prenyltransferase alpha-alpha toroid / IPR008949 [DOMAIN] - Isoprenoid synthase domain | - | magnesium ion binding; terpene synthase activity | - |
| GRMZM2G448927 | IPR017380 [FAMILY] - Histone acetyltransferase type B, catalytic subunit | chromatin silencing at telomere | histone acetyltransferase activity | chromosome, telomeric region; nucleus |
| GRMZM2G454511 | IPR001480 [DOMAIN] - Bulb-type lectin domain / IPR002290 [DOMAIN] - Serine/threonine/dual specificity protein kinase, catalytic domain | - | ATP binding; protein kinase activity | - |
| GRMZM2G472827 | - | - | - | - |
| GRMZM2G480621 | Histone-fold (IPR009072) | regulation of transcription, DNA-templated | sequence-specific DNA binding | nucleus |
| GRMZM2G509699 | - | - | - | integral component of membrane |
| GRMZM2G527017 | - | - | - | integral component of membrane |
| GRMZM2G579719 | Ribonuclease H-like domain (IPR012337) | - | nucleic acid binding | - |
| GRMZM5G804477 | - | - | - | - |
| GRMZM5G809663 | IPR009072 [DOMAIN] - Histone-fold | - | - | - |
| GRMZM5G813244 | IPR004926 [FAMILY] - Late embryogenesis abundant protein, LEA-5 | response to stress | - | - |
| GRMZM5G823824 | IPR001810 [DOMAIN] - F-box domain / IPR005174 [DOMAIN] - Domain unknown function DUF295 | - | - | - |
| GRMZM5G834759 | - | - | - | - |
| GRMZM5G839762 | - | - | - | - |
| GRMZM5G880063 | - | - | - | - |
| GRMZM5G885872 | - | - | - | - |
| GRMZM5G885938 | - | - | - | - |
| GRMZM5G887396 | - | - | - | - |
| GRMZM5G899582 | - | - | - | - |
| GRMZM2G064344 | - | - | - | integral component of membrane |
| GRMZM2G073324 | IPR001810 [DOMAIN] - F-box domain | - | - |  |
| GRMZM2G080156 | IPR001104 [DOMAIN] - 3-oxo-5-alpha-steroid 4-dehydrogenase, C-terminal | lipid metabolic process | oxidoreductase activity, acting on the CH-CH group of donors | cytoplasm; integral component of membrane |
| GRMZM2G086430 | IPR011701 [FAMILY] - Major facilitator superfamily | transmembrane transport | - | integral component of membrane |
| GRMZM2G108875 | IPR001810 [DOMAIN] - F-box domain / IPR011047 [DOMAIN] - Quinoprotein alcohol dehydrogenase-like superfamily | - | - | - |
| GRMZM2G109812 | - | - | - | - |
| GRMZM2G112942 | IPR014830 [DOMAIN] - Glycolipid transfer protein domain | - | glycolipid binding; glycolipid transporter activity | cytoplasm |
| GRMZM2G125679 | IPR011992 [DOMAIN] - EF-hand domain pair | - | calcium ion binding | integral component of membrane |
| GRMZM2G143258 | IPR029058 [DOMAIN] - Alpha/Beta hydrolase fold | - | hydrolase activity | - |
| GRMZM2G167658 | IPR011527 [DOMAIN] - ABC transporter type 1, transmembrane domain / IPR027417 [DOMAIN] - P-loop containing nucleoside triphosphate hydrolase | - | ATPase activity, coupled to transmembrane movement of substances; ATP binding | integral component of membrane |

**Supplementary Table S7. Differentially expressed genes in male inflorescence SAR^+^ relative to male inflorescence SAR^-^ using RNA-Seq data**

| **ID (maizeGDB)** | **Description** | **Base Mean** | **Log_2_ Fold Change** | ***P-value*** | ***P-adj*** |
| --- | --- | --- | --- | --- | --- |
| GRMZM2G015793 | Transferase family/N-hydroxycinnamoyl/benzoyltransferase | 113,784 | -3,373 | 4,04E-08 | 0,000154 |
| GRMZM2G021289 | Membrane protein | 78,011 | -4,007 | 1,33E-10 | 1,30E-06 |
| GRMZM2G137341 | AP2-EREBP-transcription factor 16 | 54,295 | 4,063 | 1,64E-16 | 5,01E-12 |
| GRMZM2G355572 | transposon protein CACTA | 585,537 | 2,725 | 2,34E-08 | 0,000119 |
| GRMZM2G042756 | AP2-EREBP-transcription factor 105 | 83,56 | 3,692 | 3,45E-16 | 5,27E-12 |
| GRMZM2G368838 | AP2-EREBP-transcription factor 68 | 12,196 | 3,017 | 8,54E-07 | 0,002003 |
| GRMZM2G124037 | DRE-binding protein3 | 26,402 | 3,198 | 4,74E-08 | 0,000161 |
| GRMZM2G149024 | Glycosyl transferase family 8 | 88,206 | 2,747 | 1,70E-10 | 1,30E-06 |
| GRMZM2G340257 | putative endo-1,4-beta-mannosidase family protein | 358,131 | 2,779 | 3,38E-06 | 0,006863 |
| GRMZM2G405017 | hypothetical protein | 119,271 | 1,679 | 1,67E-07 | 0,000463 |
| GRMZM2G082943 | hypothetical protein | 76,295 | -3,102 | 3,55E-08 | 0,000154 |
| GRMZM2G418343 | hypothetical protein | 773,733 | -3,072 | 3,13E-07 | 0,000796 |
| AC166636.1_FG007 | hypothetical protein | 1008,597 | -1,787 | 1,09E-07 | 0,000331 |

**Supplementary Table S8. Functional re-annotation of differentially expressed genes between mock-plants and male inflorescence SAR^+^**

| **ID (maizeGDB)** | **InterProScan annotation** | **Gene ontology**  **(biological process)** | **Gene ontology**  **(molecular function)** | **Gene ontology**  **(cellular component)** |
| --- | --- | --- | --- | --- |
| GRMZM2G015793 | IPR003480 [FAMILY] - Transferase | - | transferase activity, transferring acyl groups other than amino-acyl groups | - |
| GRMZM2G021289 | PR01217 [SPRINT] Proline rich extensin signature | - | - | - |
| GRMZM2G137341 | IPR001471 [DOMAIN] - AP2/ERF domain | transcription, DNA-templated | DNA binding; transcription factor activity, sequence-specific DNA binding | nucleus |
| GRMZM2G355572 | IPR027806 [DOMAIN] - Harbinger transposase-derived nuclease domain | - | - | - |
| GRMZM2G042756 | IPR001471 [DOMAIN] - AP2/ERF domain | transcription, DNA-templated | DNA binding; transcription factor activity, sequence-specific DNA binding | nucleus |
| GRMZM2G368838 | IPR001471 [DOMAIN] - AP2/ERF domain | transcription, DNA-templated | DNA binding; transcription factor activity, sequence-specific DNA binding | nucleus |
| GRMZM2G124037 | IPR001471 [DOMAIN] - AP2/ERF domain | transcription, DNA-templated | DNA binding; transcription factor activity, sequence-specific DNA binding | nucleus |
| GRMZM2G149024 | IPR002495 [FAMILY] - Glycosyl transferase, family 8 | - | transferase activity, transferring glycosyl groups | - |
| GRMZM2G340257 | IPR017853 [DOMAIN] - Glycoside hydrolase superfamily | carbohydrate metabolic process | - | - |
| GRMZM2G405017 | - | - | - | - |
| GRMZM2G082943 | - | - | - | integral component of membrane |
| GRMZM2G418343 | - | - | - | - |
| AC166636.1_FG007 | IPR001202 [DOMAIN] - WW domain | - | - | - |

**Supplementary Table S9. Differentially expressed genes in female inflorescence SAR^+^ relative to female inflorescence SAR^-^using RNA-Seq data**

| **ID (maizeGDB)** | **Description** | **Base Mean** | **Log_2_ Fold Change** | ***P-value*** | ***P-adj*** |
| --- | --- | --- | --- | --- | --- |
| GRMZM2G094352 | bZIP-transcription factor 107 | 235,253 | 1,79 | 1,40E-07 | 0,0002159 |
| GRMZM2G118610 | putative cinnamyl-alcohol dehydrogenase family protein | 20339,447 | 1,292 | 2,13E-05 | 0,00603618 |
| GRMZM2G474769 | LHY protein | 1105,585 | 1,338 | 7,92E-06 | 0,00327874 |
| GRMZM2G004957 | putative homeobox/lipid-binding domain family protein isoform 2 | 192,134 | -1,701 | 4,35E-05 | 0,00944939 |
| GRMZM2G099802 | anther-specific proline-rich protein APG | 462,55 | -1,438 | 6,10E-07 | 0,00064198 |
| GRMZM2G086474 | bHLH-transcription factor 134 | 56,333 | -2,491 | 4,60E-05 | 0,00984 |
| GRMZM2G016477 | putative leucine-rich repeat receptor-like protein kinase family protein | 278,494 | -2,11 | 2,52E-05 | 0,00650483 |
| GRMZM2G080516 | AP2-EREBP-transcription factor 2 | 451,049 | 1,537 | 2,38E-05 | 0,00638815 |
| GRMZM2G130079 | histone H3.2 isoform 1 | 2675,092 | -1,395 | 1,72E-07 | 0,00023792 |
| GRMZM2G013398 | C2C2-CO-like-transcription factor 6 | 445,214 | -1,119 | 2,11E-05 | 0,00603618 |
| GRMZM2G060311 | low temperature and salt responsive protein | 16,969 | -2,435 | 3,13E-05 | 0,00744908 |
| GRMZM2G128971 | fatty acid desaturase7 | 3619,021 | 1,467 | 2,84E-05 | 0,00685104 |
| GRMZM2G305046 | histone2A1 | 5583,451 | -1,265 | 4,11E-05 | 0,00916192 |
| GRMZM2G104616 | proline-rich protein | 1879,372 | -1,742 | 1,34E-05 | 0,00450163 |
| GRMZM2G376957 | histone H3.2 | 635,044 | -1,705 | 2,76E-05 | 0,00683656 |
| GRMZM2G079080 | putative argonaute family protein | 96,485 | -2,045 | 1,09E-05 | 0,00396492 |
| GRMZM2G403076 | putative FAD-binding Berberine family protein | 108,356 | 2,769 | 6,33E-12 | 4,16E-08 |
| GRMZM2G021794 | fasciclin-like arabinogalactan protein 7 | 3470,154 | 1,404 | 7,25E-06 | 0,00312538 |
| GRMZM2G441906 | grx_A2-glutaredoxin subgroup III | 71,557 | 2,322 | 3,37E-07 | 0,00042209 |
| GRMZM2G311898 | grx_I1-glutaredoxin subgroup III | 302,931 | 2,553 | 4,55E-06 | 0,00259922 |
| GRMZM2G074844 | F-box protein interaction domain containing protein | 78,143 | 2,664 | 5,58E-06 | 0,00270947 |
| GRMZM2G063896 | histone H4.3 | 2833,375 | -1,537 | 1,15E-05 | 0,00409213 |
| GRMZM2G063896 | grx_I1-glutaredoxin subgroup III | 356,943 | 2,674 | 3,14E-06 | 0,00217302 |
| GRMZM2G128215 | F-box protein interaction domain containing protein isoform 1 | 54,396 | 2,903 | 2,64E-06 | 0,0019209 |
| GRMZM2G310431 | heat shock protein1 | 2807,556 | 1,281 | 5,97E-06 | 0,00280224 |
| GRMZM2G010468 | putative cytochrome P450 superfamily protein | 1311,828 | 1,131 | 4,51E-07 | 0,00051525 |
| GRMZM2G079632 | NAC-transcription factor 4 | 328,552 | 1,077 | 4,19E-05 | 0,00921909 |
| GRMZM2G431288 | TPA: putative cytochrome P450 superfamily protein | 44,34 | 2,605 | 1,01E-05 | 0,00383919 |
| GRMZM2G165272 | HSF-transcription factor 10 | 535,689 | 0,927 | 1,78E-05 | 0,00537911 |
| GRMZM2G066528 | HMG-transcription factor 13 | 844,826 | -1,946 | 5,23E-06 | 0,00270947 |
| GRMZM2G111309 | TPA: amidase | 402,041 | 1,781 | 7,27E-10 | 2,59E-06 |
| GRMZM2G141322 | TPA: ribonuclease 1 | 30,07 | 3,191 | 2,63E-06 | 0,0019209 |
| GRMZM2G028955 | histone H2A | 1678,275 | -1,691 | 2,66E-05 | 0,00666702 |
| GRMZM2G119490 | leucine Rich Repeat family protein | 162,757 | -1,187 | 2,52E-05 | 0,00650483 |
| GRMZM2G099678 | TPA: vegetative storage protein PNI288 | 522,733 | -2,461 | 2,13E-05 | 0,00603618 |
| GRMZM2G170047 | TPA: putative cytochrome P450 superfamily protein | 115,058 | 2,061 | 8,99E-07 | 0,00084405 |
| GRMZM2G031004 | TPA: putative glycosyl hydrolase family 10 protein | 223,887 | 2,744 | 9,23E-10 | 2,59E-06 |
| AC210168.4_FG003 | TPA: putative thaumatin domain family protein | 657,888 | -2,013 | 2,48E-05 | 0,00650483 |
| GRMZM2G078314 | TPA: histone H3 | 3701,841 | -1,422 | 2,09E-05 | 0,00603618 |
| GRMZM2G148087 | WRKY-transcription factor 43 | 40,01 | 2,184 | 1,81E-08 | 3,41E-05 |
| GRMZM2G008726 | TPA: lysine decarboxylase-like protein | 39,728 | -1,939 | 2,27E-05 | 0,00619399 |
| GRMZM2G448672 | TPA: putative protein kinase superfamily protein | 77,087 | 1,698 | 3,14E-05 | 0,00744908 |
| GRMZM2G023237 | TPA: grx_I1-glutaredoxin subgroup III | 313,262 | 2,499 | 1,49E-05 | 0,00487529 |
| GRMZM2G077744 | TPA: Signal recognition particle protein isoform 2 | 855,533 | 0,98 | 8,50E-06 | 0,00338554 |
| GRMZM2G034840 | ARF-transcription factor 4 | 98,84 | -2,656 | 5,65E-06 | 0,00270947 |
| GRMZM2G421256 | MYB-related-transcription factor 20 | 817,063 | -1,832 | 3,30E-06 | 0,00222598 |
| GRMZM2G055180 | AP2-EREBP-transcription factor 198 | 365,698 | 1,988 | 3,62E-07 | 0,00043278 |
| GRMZM2G049541 | phosphoenolpyruvate carboxylase kinase4 | 324,994 | -1,209 | 1,65E-07 | 0,00023792 |
| GRMZM2G104843 | tassel seed1/ Lipoxygenase | 616,79 | 1,013 | 2,55E-05 | 0,00652215 |
| GRMZM2G447984 | TPA: histone H3.2 | 763,738 | -1,151 | 1,84E-05 | 0,00549651 |
| GRMZM2G079616 | TPA: 10-deacetylbaccatin III 10-O-acetyltransferase | 11172,689 | 0,982 | 7,98E-06 | 0,00327874 |
| GRMZM2G164074 | TPA: putative cytochrome P450 superfamily protein | 466,871 | 1,275 | 1,23E-07 | 0,00020171 |
| GRMZM2G040965 | TPA: AFH1 | 209,043 | -2,17 | 5,45E-06 | 0,00270947 |
| GRMZM2G034764 | TPA: C2 domain containing protein | 3452,999 | 1,128 | 3,61E-05 | 0,00831706 |
| GRMZM2G101409 | TPA: VQ motif family protein | 189,659 | -1,55 | 2,24E-05 | 0,00619399 |
| GRMZM2G082302 | TPA: putative RING zinc finger domain superfamily protein | 275,888 | 1,919 | 9,03E-14 | 7,92E-10 |
| GRMZM2G162829 | putative leucine-rich repeat receptor-like protein kinase family protein | 141,389 | 1,127 | 2,49E-06 | 0,0019209 |
| GRMZM2G156737 | AP2-EREBP-transcription factor 89 | 33,489 | 4,069 | 3,47E-10 | 1,52E-06 |
| GRMZM2G119071 | histone2b2 | 3571,225 | -1,586 | 1,92E-05 | 0,00566453 |
| GRMZM2G418258 | histone H3.2 | 1649,842 | -1,489 | 1,36E-05 | 0,00454122 |
| GRMZM2G151230 | cell number regulator 2 | 207,235 | 1,96 | 2,23E-09 | 5,32E-06 |
| GRMZM2G589696 | C2C2-Dof-transcription factor 43 | 64,458 | -2,291 | 2,96E-07 | 0,0003888 |
| GRMZM2G008773 | TPA: lysM domain containing protein | 145,586 | -1,541 | 7,78E-07 | 0,00075749 |
| GRMZM5G838098 | ZIM-transcription factor 27 | 786,822 | 1,25 | 1,68E-06 | 0,00142757 |
| GRMZM2G174708 | TPA: polygalacturonase inhibitor 1 | 2381,888 | 1,242 | 2,98E-09 | 6,03E-06 |
| GRMZM5G846082 | TPA: putative HLH DNA-binding domain superfamily protein | 47,64 | 2,253 | 6,30E-06 | 0,00284458 |
| GRMZM2G089528 | TPA: putative cytochrome P450 superfamily protein | 235,198 | 1,79 | 5,80E-14 | 7,63E-10 |
| GRMZM2G017087 | TPA: LOW QUALITY PROTEIN: knotted1 | 112,506 | -2,808 | 3,85E-05 | 0,0087282 |
| GRMZM2G072632 | hypothetical protein ZEAMMB73_803322 | 31,362 | 1,91 | 1,62E-05 | 0,00505743 |
| GRMZM2G018099 | hypothetical protein ZEAMMB73_801575 | 2007,124 | 1,907 | 8,71E-10 | 2,59E-06 |
| GRMZM2G157120 | hypothetical protein ZEAMMB73_804767 | 337,517 | 1,543 | 2,65E-14 | 6,97E-10 |
| GRMZM2G092256 | hypothetical protein ZEAMMB73_094227 | 905,712 | 0,974 | 5,42E-06 | 0,00270947 |
| GRMZM2G006714 | hypothetical protein ZEAMMB73_044924 | 41,486 | -2,214 | 2,18E-05 | 0,00609421 |
| GRMZM2G009025 | hypothetical protein ZEAMMB73_341562 | 29,786 | -2,656 | 4,53E-06 | 0,00259922 |
| GRMZM2G141325 | hypothetical protein | 477,877 | 3,627 | 7,71E-11 | 4,06E-07 |
| AC213621.5_FG004 | hypothetical protein | 923,905 | 1,266 | 1,98E-06 | 0,00157721 |
| AC204763.2_FG001 | hypothetical protein | 86,46 | -3,102 | 7,09E-07 | 0,0007173 |
| AC212565.3_FG001 | hypothetical protein | 3363,196 | -1,051 | 6,54E-06 | 0,0028646 |
| AC205471.4_FG008 | hypothetical protein ZEAMMB73_040603 | 73,32 | -2,667 | 2,78E-05 | 0,00683656 |
| AC204007.3_FG008 | hypothetical protein | 221,536 | -2,407 | 3,22E-05 | 0,00755721 |
| GRMZM2G162396 | TPA: hypothetical protein ZEAMMB73_544469 | 18,372 | 2,285 | 4,21E-05 | 0,00921909 |
| GRMZM2G465685 | TPA: hypothetical protein ZEAMMB73_984915 | 511,479 | 0,936 | 2,47E-05 | 0,00650483 |
| GRMZM2G318849 | TPA: hypothetical protein ZEAMMB73_835263 | 320,513 | -2,315 | 5,67E-06 | 0,00270947 |
| GRMZM2G140293 | TPA: hypothetical protein ZEAMMB73_473940 | 234,423 | 1,936 | 2,64E-05 | 0,00666665 |
| AC195147.3_FG008 | TPA: hypothetical protein ZEAMMB73_990694 | 143,734 | -2,284 | 1,78E-05 | 0,00537911 |
| GRMZM2G000825 | TPA: hypothetical protein ZEAMMB73_633017 | 109,155 | -2,429 | 3,49E-06 | 0,00223767 |
| GRMZM2G141819 | hypothetical protein ZEAMMB73_449448 | 102,405 | -2,338 | 4,97E-06 | 0,00270947 |
| GRMZM2G303419 | hypothetical protein ZEAMMB73_826452 | 38,038 | 2,287 | 3,40E-06 | 0,00223767 |
| AC207043.3_FG002 | hypothetical protein ZEAMMB73_732400 | 113,414 | 1,91 | 6,38E-06 | 0,00284458 |
| AC197355.3_FG001 | hypothetical protein ZEAMMB73_605254 | 233,73 | 2,501 | 4,48E-06 | 0,00259922 |
| GRMZM2G024561 | hypothetical protein ZEAMMB73_351842 | 37,237 | -1,852 | 2,83E-05 | 0,00685104 |
| GRMZM2G089517 | hypothetical protein ZEAMMB73_962727 | 63,818 | -2,31 | 3,49E-05 | 0,00812118 |
| GRMZM2G352274 | hypothetical protein ZEAMMB73_448179 | 239,45 | -2,566 | 1,53E-05 | 0,00489087 |
| GRMZM2G023711 | hypothetical protein ZEAMMB73_081186 | 252,42 | -1,099 | 1,46E-06 | 0,00127987 |
| GRMZM2G051005 | hypothetical protein ZEAMMB73_444294 | 1478,984 | 1,038 | 2,70E-06 | 0,0019209 |
| GRMZM2G114044 | TPA: hypothetical protein ZEAMMB73_341883 | 195,537 | 1,351 | 6,21E-06 | 0,00284458 |
| GRMZM2G077227 | TPA: hypothetical protein ZEAMMB73_402135 | 158,139 | -2,368 | 2,28E-05 | 0,00619399 |
| GRMZM2G166064 | TPA: hypothetical protein ZEAMMB73_426606 | 389,463 | 1,222 | 1,07E-05 | 0,00396492 |
| GRMZM2G395983 | TPA: hypothetical protein ZEAMMB73_364972 | 151,333 | -2,256 | 9,60E-06 | 0,00376949 |
| GRMZM2G062245 | TPA: hypothetical protein ZEAMMB73_460101 | 59,47 | -2,343 | 1,00E-05 | 0,00383919 |
| GRMZM2G027478 | TPA: hypothetical protein ZEAMMB73_793304 | 298,571 | 1,147 | 3,71E-05 | 0,00848585 |
| GRMZM5G874955 | TPA: hypothetical protein ZEAMMB73_940685 | 742,7 | 1,615 | 8,22E-06 | 0,00332702 |
| GRMZM2G065989 | TPA: hypothetical protein ZEAMMB73_742097 | 179,154 | -1,52 | 1,04E-05 | 0,00392151 |
| GRMZM2G071119 | TPA: hypothetical protein ZEAMMB73_781870, partial | 1194,227 | -0,947 | 5,10E-06 | 0,00270947 |
| GRMZM2G303118 | TPA: hypothetical protein ZEAMMB73_446301 | 274,525 | -2,353 | 9,85E-10 | 2,59E-06 |
| AC191961.3_FG009 | TPA: hypothetical protein ZEAMMB73_667658, partial | 50,016 | -2,206 | 4,09E-05 | 0,00916192 |
| GRMZM2G361100 | TPA: hypothetical protein ZEAMMB73_587734 | 438,036 | 1,04 | 3,67E-06 | 0,00224173 |
| GRMZM2G062504 | TPA: hypothetical protein ZEAMMB73_923611 | 50,503 | -2,901 | 1,23E-05 | 0,0042034 |
| GRMZM2G475536 | TPA: hypothetical protein ZEAMMB73_851082 | 55,843 | -2,842 | 2,48E-09 | 5,43E-06 |
| GRMZM2G409722 | TPA: hypothetical protein ZEAMMB73_771474 | 387,15 | 1,559 | 4,73E-07 | 0,0005181 |
| GRMZM2G172945 | TPA: hypothetical protein ZEAMMB73_046692 | 71,354 | -2,467 | 1,14E-05 | 0,0040901 |
| GRMZM2G004180 | hypothetical protein ZEAMMB73_460990 | 41,215 | -2,649 | 3,57E-06 | 0,00223767 |
| GRMZM2G061303 | hypothetical protein ZEAMMB73_688553 | 42,837 | 3,292 | 4,87E-08 | 8,54E-05 |
| GRMZM2G116640 | hypothetical protein ZEAMMB73_865081 | 61,002 | 2,03 | 4,49E-05 | 0,00967396 |
| GRMZM2G398603 | TPA: hypothetical protein ZEAMMB73_375988 | 74,298 | 2,338 | 1,60E-05 | 0,00505743 |
| GRMZM2G160466 | TPA: hypothetical protein ZEAMMB73_908552 | 108,316 | -2,269 | 1,17E-05 | 0,00409213 |
| GRMZM2G062458 | TPA: hypothetical protein ZEAMMB73_396669 | 40,45 | 2,354 | 1,50E-05 | 0,00487529 |
| GRMZM2G141320 | TPA: hypothetical protein ZEAMMB73_675403 | 57,122 | -2,707 | 5,10E-06 | 0,00270947 |
| GRMZM6G132224 | locus record not found | 128,862 | 2,816 | 1,21E-06 | 0,00109564 |
| GRMZM2G180246 | TPA: hypothetical protein ZEAMMB73_811390 | 272,687 | -2,631 | 1,84E-06 | 0,00151332 |
| GRMZM2G120440 | TPA: hypothetical protein ZEAMMB73_368815 | 87,305 | -2,477 | 1,20E-05 | 0,00414258 |
| GRMZM6G778611 | locus record not found | 198,498 | -2,329 | 1,71E-05 | 0,00528874 |
| GRMZM6G738249 | locus record not found | 91,206 | 2,295 | 7,41E-06 | 0,00314141 |

**Supplementary Table S10. Functional re-annotation of differentially expressed genes between mock-plants and female inflorescence SAR^+^**

| **ID (maizeGDB)** | **InterProScan annotation** | **Gene ontology**  **(biological process)** | **Gene ontology**  **(molecular function)** | **Gene ontology**  **(cellular component)** |
| --- | --- | --- | --- | --- |
| GRMZM2G094352 | IPR004827 [DOMAIN] - Basic-leucine zipper domain | transcription, DNA-templated | sequence-specific DNA binding; transcription factor activity, sequence-specific DNA binding | - |
| GRMZM2G118610 | IPR013149 [DOMAIN] - Alcohol dehydrogenase, C-terminal | - | oxidoreductase activity; zinc ion binding | - |
| GRMZM2G474769 | - | circadian rhythm; long-day photoperiodism, flowering; negative regulation of circadian rhythm; negative regulation of sequence-specific DNA binding transcription factor activity; response to cold;response to red or far red light | transcription factor activity, sequence-specific DNA binding; transcription regulatory region DNA binding | - |
| GRMZM2G004957 | IPR009057 [DOMAIN] - Homeodomain-like | regulation of transcription, DNA-templated | lipid binding; sequence-specific DNA binding | nucleus |
| GRMZM2G099802 | IPR013830 [DOMAIN] - SGNH hydrolase-type esterase domain | - | hydrolase activity, acting on ester bonds | - |
| GRMZM2G086474 | IPR011598 [DOMAIN] - Myc-type, basic helix-loop-helix (bHLH) domain | - | - | - |
| GRMZM2G016477 | IPR013210 [DOMAIN] - Leucine-rich repeat-containing N-terminal, plant-type | - | ATP binding; protein kinase activity | integral component of membrane |
| GRMZM2G080516 | IPR001471 [DOMAIN] - AP2/ERF domain | transcription, DNA-templated | DNA binding; transcription factor activity, sequence-specific DNA binding | nucleus |
| GRMZM2G130079 | IPR007125 [DOMAIN] - Histone H2A/H2B/H3 | cell proliferation | DNA binding | nucleosome; nucleus |
| GRMZM2G013398 | IPR000315 [DOMAIN] - B-box-type zinc finger | - | zinc ion binding | intracellular |
| GRMZM2G060311 | IPR000612 [FAMILY] - Proteolipid membrane potential modulator | - | - | integral component of membrane |
| GRMZM2G128971 | IPR021863 [DOMAIN] - Fatty acid desaturase, N-terminal | lipid metabolic process | oxidoreductase activity, acting on paired donors, with oxidation of a pair of donors resulting in the reduction of molecular oxygen to two molecules of water | integral component of membrane |
| GRMZM2G305046 | IPR002119 [FAMILY] - Histone H2A | - | DNA binding | nucleosome; nucleus |
| GRMZM2G104616 | - | - | - | - |
| GRMZM2G376957 | IPR009072 [DOMAIN] - Histone-fold | cell proliferation | DNA binding | nucleosome; nucleus |
| GRMZM2G079080 | IPR032474 [DOMAIN] - Protein argonaute, N-terminal | - | nucleic acid binding | - |
| GRMZM2G403076 | IPR016167 [DOMAIN] - FAD-binding, type 2, subdomain 1 | - | flavin adenine dinucleotide binding;oxidoreductase activity, acting on CH-OH group of donors | - |
| GRMZM2G021794 | IPR000782 [DOMAIN] - FAS1 domain | - | - | - |
| GRMZM2G441906 | IPR002109 [DOMAIN] - Glutaredoxin | cell redox homeostasis | electron carrier activity; protein disulfide oxidoreductase activity | cell |
| GRMZM2G311898 | IPR002109 [DOMAIN] - Glutaredoxin | cell redox homeostasis | electron carrier activity; protein disulfide oxidoreductase activity | cell |
| GRMZM2G074844 | IPR001810 [DOMAIN] - F-box domain | - | - | - |
| GRMZM2G063896 | IPR001951 [FAMILY] - Histone H4 | nucleosome assembly | DNA binding | nucleosome; nucleus; chloroplast; cytosol; nucleolus; plasma membrane;plasmodesma; thylakoid; vacuolar membrane |
| GRMZM2G063896 | IPR002109 [DOMAIN] - Glutaredoxin | - | - | - |
| GRMZM2G128215 | IPR001810 [DOMAIN] - F-box domain | - | - | - |
| GRMZM2G310431 | IPR029047 [DOMAIN] - Heat shock protein 70kD, peptide-binding domain | - | ATP binding | - |
| GRMZM2G010468 | IPR001128 [FAMILY] - Cytochrome P450 | - | heme binding; iron ion binding; monooxygenase activity; oxidoreductase activity, acting on paired donors, with incorporation or reduction of molecular oxygen | - |
| GRMZM2G079632 |  | regulation of transcription, DNA-templated; transcription | DNA binding | nucleus |
| GRMZM2G431288 | IPR001128 [FAMILY] - Cytochrome P450 | - | heme binding; iron ion binding;monooxygenase activity; oxidoreductase activity, acting on paired donors, with incorporation or reduction of molecular oxygen | - |
| GRMZM2G165272 | IPR000232 [DOMAIN] - Heat shock factor (HSF)-type, DNA-binding | - | sequence-specific DNA binding; transcription factor activity, sequence-specific DNA binding | nucleus |
| GRMZM2G066528 | IPR009071 [DOMAIN] - High mobility group box domain | - | - | - |
| GRMZM2G111309 | IPR023631 [DOMAIN] - Amidase signature domain | - | carbon-nitrogen ligase activity, with glutamine as amido-N-donor | - |
| GRMZM2G141322 | IPR001568 [FAMILY] - Ribonuclease T2-like | - | ribonuclease T2 activity; RNA binding | - |
| GRMZM2G028955 | IPR002119 [FAMILY] - Histone H2A | - | DNA binding | nucleosome; nucleus |
| GRMZM2G119490 | IPR032675 [DOMAIN] - Leucine-rich repeat domain, L domain-like | - | - | - |
| GRMZM2G099678 | IPR000845 [DOMAIN] - Nucleoside phosphorylase domain | nucleoside metabolic process | catalytic activity | integral component of membrane |
| GRMZM2G170047 | IPR001128 [FAMILY] - Cytochrome P450 | - | heme binding; iron ion binding;monooxygenase activity; oxidoreductase activity, acting on paired donors, with incorporation or reduction of molecular oxygen | - |
| GRMZM2G031004 | IPR001000 [FAMILY] - Glycoside hydrolase, family 10 | carbohydrate metabolic process | hydrolase activity, hydrolyzing O-glycosyl compounds | - |
| AC210168.4_FG003 | IPR001938 [FAMILY] - Thaumatin | - | - | - |
| GRMZM2G078314 | IPR007125 [DOMAIN] - Histone H2A/H2B/H3 | - | DNA binding | nucleosome; nucleus |
| GRMZM2G148087 | IPR003657 [DOMAIN] - WRKY domain | - | sequence-specific DNA binding; transcription factor activity, sequence-specific DNA binding | - |
| GRMZM2G008726 | IPR031100 [FAMILY] - LOG family | - | - | integral component of membrane |
| GRMZM2G448672 | IPR013320 [DOMAIN] - Concanavalin A-like lectin/glucanase domain / IPR002290 [DOMAIN] - Serine/threonine/dual specificity protein kinase, catalytic domain | - | ATP binding; protein kinase activity | - |
| GRMZM2G023237 | IPR002109 [DOMAIN] - Glutaredoxin | cell redox homeostasis | electron carrier activity; protein disulfide oxidoreductase activity | cell |
| GRMZM2G077744 | IPR002778 [FAMILY] - Signal recognition particle, SRP19 subunit | SRP-dependent cotranslational protein targeting to membrane | 7S RNA binding | signal recognition particle |
| GRMZM2G034840 | IPR010525 [DOMAIN] - Auxin response factor | auxin-activated signaling pathway; regulation of transcription, DNA-templated; transcription | DNA binding | nucleus |
| GRMZM2G421256 | IPR006447 [DOMAIN] - Myb domain, plants | regulation of transcription, DNA-templated; transcription | DNA binding | nucleus |
| GRMZM2G055180 | IPR001471 [DOMAIN] - AP2/ERF domain | transcription, DNA-templated | DNA binding; transcription factor activity, sequence-specific DNA binding | nucleus |
| GRMZM2G049541 | IPR011009 [DOMAIN] - Protein kinase-like domain | - | ATP binding; protein kinase activity | - |
| GRMZM2G104843 | IPR001246 [FAMILY] - Lipoxygenase, plant | oxylipin biosynthetic process | metal ion binding; oxidoreductase activity, acting on single donors with incorporation of molecular oxygen, incorporation of two atoms of oxygen | - |
| GRMZM2G447984 | IPR000164 [FAMILY] - Histone H3/CENP-A | - | DNA binding | nucleosome; nucleus |
| GRMZM2G079616 | IPR023213 [DOMAIN] - Chloramphenicol acetyltransferase-like domain | - | transferase activity, transferring acyl groups other than amino-acyl groups | - |
| GRMZM2G164074 | IPR001128 [FAMILY] - Cytochrome P450 | - | heme binding; iron ion binding;monooxygenase activity; oxidoreductase activity, acting on paired donors, with incorporation or reduction of molecular oxygen | - |
| GRMZM2G040965 | IPR015425 [DOMAIN] - Formin, FH2 domain | - | - | integral component of membrane |
| GRMZM2G034764 | IPR000008 [DOMAIN] - C2 domain | - | - | - |
| GRMZM2G101409 | IPR008889 [DOMAIN] - VQ | - | - | - |
| GRMZM2G082302 | IPR013083 [DOMAIN] - Zinc finger, RING/FYVE/PHD-type | - | metal ion binding; ubiquitin-protein transferase activity | - |
| GRMZM2G162829 | IPR013210 [DOMAIN] - Leucine-rich repeat-containing N-terminal, plant-type | - | ATP binding; protein serine/threonine kinase activity | integral component of membrane |
| GRMZM2G156737 | IPR001471 [DOMAIN] - AP2/ERF domain | transcription, DNA-templated | DNA binding; transcription factor activity, sequence-specific DNA binding | nucleus |
| GRMZM2G119071 | IPR000558 [FAMILY] - Histone H2B | - | DNA binding | nucleosome; nucleus |
| GRMZM2G418258 | IPR000164 [FAMILY] - Histone H3/CENP-A | cell proliferation | DNA binding | nucleosome; nucleus |
| GRMZM2G151230 | IPR006461 [FAMILY] - PLAC8 motif-containing protein | negative regulation of cell proliferation | - | integral component of membrane |
| GRMZM2G589696 | IPR003851 [DOMAIN] - Zinc finger, Dof-type | regulation of transcription, DNA-templated | DNA binding | - |
| GRMZM2G008773 | IPR018392 [DOMAIN] - LysM domain | - | - | integral component of membrane |
| GRMZM5G838098 | IPR010399 [DOMAIN] - Tify domain | - | - | - |
| GRMZM2G174708 | IPR032675 [DOMAIN] - Leucine-rich repeat domain, L domain-like | - | - | - |
| GRMZM5G846082 | - | - | - | - |
| GRMZM2G089528 | IPR001128 [FAMILY] - Cytochrome P450 | - | heme binding; iron ion binding; monooxygenase activity; oxidoreductase activity, acting on paired donors, with incorporation or reduction of molecular oxygen | integral component of membrane |
| GRMZM2G017087 |  | regulation of transcription, DNA-templated; transcription | sequence-specific DNA binding | nucleus |
| GRMZM2G072632 | IPR004776 [FAMILY] - Auxin efflux carrier | transmembrane transport | - | integral component of membrane |
| GRMZM2G018099 | - | - | - | - |
| GRMZM2G157120 | - | - | - | - |
| GRMZM2G092256 | IPR009943 [FAMILY] - Protein of unknown function DUF1475 | - | - | integral component of membrane |
| GRMZM2G006714 | IPR012416 [FAMILY] - CALMODULIN-BINDING PROTEIN60 | response to stress | - | - |
| GRMZM2G009025 | IPR001701 [FAMILY] - Glycoside hydrolase, family 9 | cellulose catabolic process | cellulase activity | - |
| GRMZM2G141325 | - | - | - | - |
| AC213621.5_FG004 | IPR000782 [DOMAIN] - FAS1 domain | - | - | - |
| AC204763.2_FG001 | IPR032675 [DOMAIN] - Leucine-rich repeat domain, L domain-like | - | - | - |
| AC212565.3_FG001 | IPR001951 [FAMILY] - Histone H4 | nucleosome assembly | DNA binding | nucleosome; nucleus |
| AC205471.4_FG008 | IPR032675 [DOMAIN] - Leucine-rich repeat domain, L domain-like | - | - | - |
| AC204007.3_FG008 | - | - | - | integral component of membrane |
| GRMZM2G162396 | IPR008480 [FAMILY] - Protein of unknown function DUF761, plant | - | - | integral component of membrane |
| GRMZM2G465685 | - | - | - | - |
| GRMZM2G318849 | IPR016024 [DOMAIN] - Armadillo-type fold | cell proliferation; chromatin silencing by small RNA; DNA replication initiation; histone H3-K9 methylation; methylation-dependent chromatin silencing; microtubule-based process;regulation of cell cycle; regulation of DNA replication; regulation of flower development |  | - |
| GRMZM2G140293 | - | - | - | - |
| AC195147.3_FG008 | - | - | - | - |
| GRMZM2G000825 | IPR011990 [DOMAIN] - Tetratricopeptide-like helical domain | - | - | - |
| GRMZM2G141819 | - | - | - | - |
| GRMZM2G303419 | IPR005299 [FAMILY] - SAM dependent carboxyl methyltransferase | - | methyltransferase activity | - |
| AC207043.3_FG002 | IPR008889 [DOMAIN] - VQ | - | - | - |
| AC197355.3_FG001 | - | - | - | - |
| GRMZM2G024561 | IPR012946 [DOMAIN] - X8 domain | - | - | - |
| GRMZM2G089517 | - | - | - | - |
| GRMZM2G352274 | - | - | - | - |
| GRMZM2G023711 | IPR005746 [FAMILY] - Thioredoxin | cell redox homeostasis; glycerol ether metabolic process | protein disulfide oxidoreductase activity | cell |
| GRMZM2G051005 | IPR023213 [DOMAIN] - Chloramphenicol acetyltransferase-like domain | - | transferase activity, transferring acyl groups other than amino-acyl groups | - |
| GRMZM2G114044 | IPR018790 [FAMILY] - Protein of unknown function DUF2358 | - | - | - |
| GRMZM2G077227 | - | - | - | - |
| GRMZM2G166064 | IPR025064 [DOMAIN] - Domain of unknown function DUF4005 | - | - | - |
| GRMZM2G395983 | IPR008480 [FAMILY] - Protein of unknown function DUF761, plant | - | - | - |
| GRMZM2G062245 | - | - | - | - |
| GRMZM2G027478 | IPR008013 [DOMAIN] - GATA-type transcription activator, N-terminal | positive regulation of transcription, DNA-templated | DNA binding; zinc ion binding | nucleus |
| GRMZM5G874955 | IPR003439 [DOMAIN] - ABC transporter-like | - | ATPase activity; ATP binding | integral component of membrane |
| GRMZM2G065989 | IPR031142 [FAMILY] - SPX domain-containing protein | cellular response to phosphate starvation | - | - |
| GRMZM2G071119 | IPR001807 [FAMILY] - Chloride channel, voltage gated | - | voltage-gated chloride channel activity | integral component of membrane |
| GRMZM2G303118 | IPR027417 [DOMAIN] - P-loop containing nucleoside triphosphate hydrolase | - | - | - |
| AC191961.3_FG009 | IPR027417 [DOMAIN] - P-loop containing nucleoside triphosphate hydrolase | - | - | - |
| GRMZM2G361100 | IPR003613 [DOMAIN] - U box domain | - | ligase activity; ubiquitin-protein transferase activity | - |
| GRMZM2G062504 | - | - | - | - |
| GRMZM2G475536 | - | - | - | - |
| GRMZM2G409722 | - | - | - | - |
| GRMZM2G172945 | - | - | - | - |
| GRMZM2G004180 | IPR008480 [FAMILY] - Protein of unknown function DUF761, plant | - | - | integral component of membrane |
| GRMZM2G061303 | IPR000109 [FAMILY] - Proton-dependent oligopeptide transporter family | - | transporter activity | integral component of membrane |
| GRMZM2G116640 | IPR003406 [FAMILY] - Glycosyl transferase, family 14 | - | acetylglucosaminyltransferase activity | membrane |
| GRMZM2G398603 | IPR003604 [DOMAIN] - Zinc finger, U1-type / IPR002885 [REPEAT] - Pentatricopeptide repeat | - | acetylglucosaminyltransferase activity | membrane |
| GRMZM2G160466 | - | - | nucleic acid binding; zinc ion binding | - |
| GRMZM2G062458 | - | - | - | - |
| GRMZM2G141320 | IPR009695 [DOMAIN] - Diacylglycerol glucosyltransferase, N-terminal | glycolipid biosynthetic process | transferase activity, transferring hexosyl groups | - |
| GRMZM6G132224 | IPR007493 [FAMILY] - Protein of unknown function DUF538 | - | - | nucleus |
| GRMZM2G180246 | IPR007726 [FAMILY] - SS18 family | adaxial/abaxial pattern specification; cell proliferation; leaf morphogenesis; ovule development | transcription coactivator activity | - |
| GRMZM2G120440 | - | - | - | - |
| GRMZM6G778611 | IPR001251 [DOMAIN] - CRAL-TRIO lipid binding domain | cell wall pectin metabolic process; plant-type cell wall cellulose metabolic process; sterol biosynthetic process; transport | - | cytosol; integral component of membrane; nucleus; plasma membrane |
| GRMZM6G738249 | IPR003337 [FAMILY] - Trehalose-phosphatase | trehalose biosynthetic process | trehalose-phosphatase activity | - |

1. ^1^Centro de Análises Proteômicas e Bioquímicas, Pós-Graduação em Ciências Genômicas e Biotecnologia Universidade Católica de Brasília, Brasília-DF, Brazil. ^2^Fundação Oswaldo Cruz, Fiocruz, Belo Horizonte, Brazil. ^3^Research Laboratory of Electronics - Massachusetts Institute of Technology (MIT), Cambridge, MA, USA. ^4^Embrapa Recursos Genéticos e Biotecnologia, Brasília, DF, Brazil. ^5^Embrapa Milho e Sorgo, Sete Lagoas, Brazil. ^6^S-Inova Biotech, Pos-Graduação em Biotecnologia, Universidade Católica Dom Bosco, Campo Grande, Brazil. ^7^Instituto Federal de Brasília, DF, Brazil. *Corresponding author: ocfranco@gmail.com (OLF). [↑](#footnote-ref-2)
